# Microbial response to single-cell protein production and brewery wastewater treatment

**DOI:** 10.1111/1751-7915.12128

**Published:** 2014-05-16

**Authors:** Jackson Z Lee, Andrew Logan, Seth Terry, John R Spear

**Affiliations:** 1 Department of Civil and Environmental Engineering, Colorado School of MinesGolden, CO, USA; 2 Nutrinsic, Corp.Aurora, CO, USA

## Abstract

As global fisheries decline, microbial single-cell protein (SCP) produced from brewery process water has been highlighted as a potential source of protein for sustainable animal feed. However, biotechnological investigation of SCP is difficult because of the natural variation and complexity of microbial ecology in wastewater bioreactors. In this study, we investigate microbial response across a full-scale brewery wastewater treatment plant and a parallel pilot bioreactor modified to produce an SCP product. A pyrosequencing survey of the brewery treatment plant showed that each unit process selected for a unique microbial community. Notably, flow equalization basins were dominated by *Prevotella*, methanogenesis effluent had the highest levels of diversity, and clarifier wet-well samples were sources of sequences for the candidate bacterial phyla of TM7 and BD1-5. Next, the microbial response of a pilot bioreactor producing SCP was tracked over 1 year, showing that two different production trials produced two different communities originating from the same starting influent. However, SCP production resulted generally in enrichment of several clades of rhizospheric diazotrophs of *Alphaproteobacteria* and *Betaproteobacteria* in the bioreactor and even more so in the final product. These diazotrophs are potentially useful as the basis of a SCP product for commercial feed production.

## Introduction

Already half of all global fish stocks have been deemed fully exploited (Cressey, 2009), which has led to the collapse of several fisheries and the potential collapse of others over the next several decades (Worm *et al*., 2006). Concomitantly, aquaculture (the farm rearing of fish) has grown at an annual rate of 14% since 1970 (FAO Fisheries Department, 2003). Because aquaculture feed production relies on significant amounts of non-sustainable fish meal protein harvested from ocean fisheries, further aquaculture growth will result in more fish meal shortages and further depletion of ocean fisheries. Therefore, there has been renewed interest in the development of less expensive and more sustainable fish meal replacements.

In the brewing industry, solid byproducts of various forms (spent grains, hops, yeasts, etc.), once a costly landfill waste, have become a livestock feed source. Even after this removal of solids, a large amount of dissolved carbon still remains in the typical brewery wastewater (Hough, 1985). This brewery waste can be aerobically and microbiologically treated in a process-wastewater treatment facility and the carbon-degrading microbiota harvested as dried microbial biomass, called single-cell protein (SCP). Researchers have recognized SCP's potential as an animal feed for decades, although SCP has never fully replaced fish meal at production scale (El-Sayed, 1999). A major concern has been the negative performance and connotations associated with wastewater and, in particular, reuse of raw sewage. Initial studies in SCP production from domestic wastewater biomass sources were often plagued by heavy metal contamination and faecal pathogens as part of their process stream (Tacon, 1979; Lovell, 1989). However, food-processing wastes have minor or no exposure to domestic sewage (Vriens *et al*., 1989) and are known to have a much higher chemical oxygen demand (COD) and lower total nitrogen profile than domestic sewage (Gray, 2004). Specifically, brewery process water possesses distinct characteristics that make the technology more feasible than most food-processing process water types, such as a continuous global year-round production of dissolved carbon process water (Huige, 2006), and an amino acid profile rich in lysine and methionine (two essential amino acids absent from many plant and fungal sources) that is comparable with fish meal (Vriens *et al*., 1989; Tacon *et al*., 2009). Several major challenges to bring SCP to market are to reliably maintain a high crude protein and essential amino acid content, and to continue to produce a treated process-wastewater that meets local water regulations. An understanding of the underlying microbial community responsible for SCP product formation is needed to help achieve these goals.

Fortuitously, knowledge of microbial communities is enabled by the rapid increase in DNA sequencing throughput with decreased cost, which has greatly expanded the detection coverage of microbial diversity in environmental samples and has the potential to identify the functioning and turnover of species in environmental engineering applications such as wastewater technology. Several recent studies have established that high-throughput sequencing can track how wastewater community consortia fluctuate in real-world systems (Werner *et al*., 2011) and how wastewater treatment diversity can have notable biogeographical trends between plants (Werner *et al*., 2011; Zhang *et al*., 2012). The production of novel bioproducts from wastewater such as fuel and chemicals requires a closer look at how these microbial systems function in order to identify relevant communities responsible for mixed community biotechnologies [e.g. microbial fuel cells (Lee *et al*., 2010), and lignin-cellulose degradation (Hollister *et al*., 2011)]. In this study, the microbial turnover and diversity of an entire brewery wastewater treatment works was analysed by pyrotag sequencing technology in order to identify metabolisms of biotechnological interest and to develop possible strategies to improve quality or economic competitiveness. Specifically, this study attempted to identify what common microbial community responses were seen in an SCP production pilot bioreactor running under a non-conventional aerobic treatment regime and the relationship of this community compared with the microbial consortia of the larger treatment plant facility.

## Results

### Physical characteristics and operational conditions of the wastewater treatment facility

Table 1 shows the operational conditions of unit processes in this study. For the wastewater treatment facility, operational parameters were tracked for each stage by plant operators. This data indicated high COD wastewaters and a sizable suspended solids fraction in both the influent and in the acidogenic basins. Organic acid and volatile fatty acids (VFA) monitoring of the basin indicated production of organic acids and (in conjunction with free ammonia measurements) also indicated a relatively low ratio of free nitrogen to carbon available for microbes. In the methanogenic upflow anaerobic sludge blanket (UASB) basin, further acid production was noted, and the majority of this was consumed in the UASB with a notable sludge blanket observable as total suspended solids (TSS). These constituents were then aerobically treated to discharge standards in the aerobic basin. In contrast, the pilot bioreactor was marked by a higher mixed liquor suspended solids (MLSS) content than most conventional domestic wastewater treatment regimes, as well as much lower dissolved oxygen (DO) levels and higher VFA levels due to influent from the acidogenic basin.

**Table 1:** Operating parameters of each unit process

Sample location	Operating conditions	Average (SD)	
Plant influent sample	Flow rate	402 (174)	m^3^ d^−1^
	pH	10.85 (1.3)	
	COD total	10 147 (3848)	mg l^−1^
	COD soluble	7859 (2573)	mg l^−1^
	TSS	1989 (1011)	mg l^−1^
	Temperature	35	°C
Acidogenic basin mixed liquor	Volume	600	m^3^
	pH	5.68 (0.43)	
	VFA	1654 (284)	mg l^−1^ acetic acid
	Total organic acids	4489 (1439)	mg l^−1^
	COD total	10 554 (1854)	mg l^−1^
	COD soluble	8005 (1714)	mg l^−1^
	TSS	1275 (352)	mg l^−1^
	Total N	164 (18)	mg l^−1^
	P-PO_4_	152 (21)	mg l^−1^
	NH_3_-N	8.76 (9.48)	mg l^−1^
Methanogenesis UASB outfall	Volume	800	m^3^
	VFA	4418 (2567)	mg l^−1^ acetic acid
	TSS	4705 (2785)	mg l^−1^
Aerobic basin mixed liquor	Volume	3820	m^3^
	SRT	16	d
Clarifier outfall	COD total	131 (22)	mg l^−1^
	COD soluble	123 (22)	mg l^−1^
	TSS	12 (5)	mg l^−1^
Pilot bioreactor sample port	Volume	38	m^3^
	Flow rate	4.75	m^3^ d^−1^
	pH	7.15 (0.36)	
	MLSS	2600 (461)	mg l^−1^
	Total organic acids	3666 (1301)	mg l^−1^
	DO	0.22 (0.05)	mg l^−1^
	Excess N, P added as urea and phosphoric acid		

SD, standard deviation; SRT, solids retention time.

### Pyrotag microbial diversity across the wastewater treatment plant

Table 2 shows alpha diversity information and sequencing depth for each sample. A total of 808 near full-length Sanger and a total of ∼ 54 000 pyrotag 16S amplicon sequences were completed. Microbial diversity coverage estimates based on alpha diversity were bracketed on the low end by the Chao1 estimator and on the high end by the CatchAll estimate. Chao1 and abundance-based coverage estimator (ACE) metrics of samples rarified to the same sequencing depth showed that the influent and acidogenic basin had the lowest levels of diversity, while the majority of operational taxonomic units (OTUs) in this study were found from both the UASB bioreactor and the aerobic basin of the brewery wastewater treatment plant (WWTP). Figure 2 describes phylum-level diversity from all samples arranged by unweighted pair group method with arithmetic mean (UPGMA) cladogram based on the unweighted UniFrac distance, which shows that samples clustered primarily because of unit process type (jackknife sensitivity analysis of sequencing depth in Supporting Information Fig. S1). Samples tended to primarily cluster by treatment regime, except the pilot reactor mixed liquor samples clustered closely to the corresponding time point of the final dried SCP product. The four main dominant phyla found from pyrotags in this study were the *Bacteroidetes*, *Firmicutes*, *Actinobacteria* and *Proteobacteria* (from the alpha, beta and gamma classes), and were consistent with previous research on wastewater aerobic treatment (Seviour and Nielsen, 2010) and process wastewater specifically (Manz *et al*., 1994). The candidate divisions of SR1 and RF3, seen previously in methanogenesis bioreactor surveys (Chouari *et al*., 2005; Riviere *et al*., 2009), comprised 2.8% and 2.3% of sequences respectively from the methanogenesis UASB process. TM7 (Hugenholtz *et al*., 2001) and BD1-5 (Li *et al*., 1999) accounted for 5.2% and 5.1% of sequences detected in the treatment plant wet well. Krona hierarchical pie charts (Supplemental Information, http://inside.mines.edu/~jspear/resources.html) showed the relative abundance of OTUs across lower taxonomic levels. The influent was dominated by several groups of *Firmicutes* from the *Lactobacillus* and *Enterococcus* families. Acidogenic basin pyrotag sequences were dominated by the genus *Prevotella*, a saccharolytic fermenter. The remaining stages showed no highly dominant clades across time, although several clades of closely related enriched OTUs (from *Bacteroidetes*, *Firmicutes* and *Proteobacteria*) appear to dominate in the pilot bioreactor and SCP product. In addition, approximately 14.3% of all OTUs (representing 4% of total sequences) remained unclassified beyond the domain level (5.9% of pilot plant OTUs, 5.2% of methanogenesis OTUs and 4.0% of aerobic basin OTUs).

**Table 2:** Sampling schedule, sequencing depth and alpha diversity of each unit process

						Alpha diversity estimator	
Sample location	Date, day number	Number of Sanger reads	Number of pyrotags (V1-V2)	Observed OTUs (97%)	% coverage	Chao1 Average (SD)^a^	ACE Average (SD)^a^
Plant influent sample port	1/30/08, 0	–	459	52	46–76	68.2	76.0
	3/19/08, 49	–	816	38	54–66	48.4 (11.8)	54.0 (12.2)
	4/30/09, 456	–	674	8	56–84	8.8 (2.0)	10.2 (2.6)
Acidogenic basin mixed liquor	1/30/08, 0	–	1127	38	70–73	39.4 (11.0)	43.4 (10.8)
	3/19/08, 49	92	3162	85	45–58	66.1 (16.5)	71.2 (15.2)
	3/19/09, 414	52	3450	66	67–93	51.4 (12.1)	52.5 (7.3)
	4/30/09, 456	43	884	41	58–76	48.4 (12.9)	50.1 (10.5)
Methanogenesis UASB outfall	1/30/08, 0	–	975	128	51–60	159.8 (23.2)	172.4 (21.4)
	3/19/08, 49	14	2373	220	59–71	193.5 (31.5)	191.8 (27.0)
	4/30/09, 456	72	805	131	32–58	192.1 (25.1)	192.2 (18.3)
Aerobic basin mixed liquor	4/30/09, 456	34	1097	194	45–48	255.4 (38.7)	267.5 (30.2)
	4/30/09, 456	–	722	155	43–65	222.8 (29.9)	239.7 (22.4)
	4/30/09, 456	–	1226	211	46–63	266.2 (42.4)	275.8 (37.4)
Clarifier outfall	1/30/08, 0	–	1176	138	40–59	174.9 (46.7)	193.8 (32.3)
	3/19/08, 49	52	2439	315	53–61	276.2 (41.7)	305.6 (45.0)
	4/30/09, 456	38	960	176	45–66	220.3 (25.9)	223.8 (21.8)
Pilot bioreactor sample port	03/19/08, 49	48	4090	88	52–71	68.6 (30.7)	71.0 (16.7)
	04/08/08, 69	–	1217	127	39–63	167.8 (34.2)	177.3 (27.7)
	09/10/08, 224	24	4874	197	59–71	127.8 (31.1)	136.2 (26.6)
	01/06/09, 342	29	2840	193	50–70	163.2 (25.4)	164.1 (21.9)
	02/16/09, 383	56	3005	163	53–75	130.9 (32.1)	133.7 (25.9)
	03/19/09, 414	51	1711	151	63–70	159.2 (26.2)	165.2 (19.1)
	04/30/09, 456	25	1231	115	33–59	137.5 (28.1)	143.9 (25.8)
Dried SCP product	05/07/08, 98	46	3266	92	63–79	72.4 (13.4)	78.9 (12.8)
	09/10/08, 224	19	3580	123	55–70	90.3 (19.1)	102.3 (19.8)
	01/06/09, 342	41	2122	171	64–74	160.5 (25.9)	171.1 (24.3)
	02/16/09, 383	–	1309	103	52–71	119.2 (22.4)	128.3 (23.8)
	03/19/09, 414	37	2262	147	56–68	142.2 (32.0)	152.9 (28.7)
	04/30/09, 456	35	1104	94	46–60	118.8 (22.9)	123.9 (18.6)

a Jackknifed to 500 sequences sample^–1^ for all samples except first.

SD, standard deviation.

**Fig 1:**
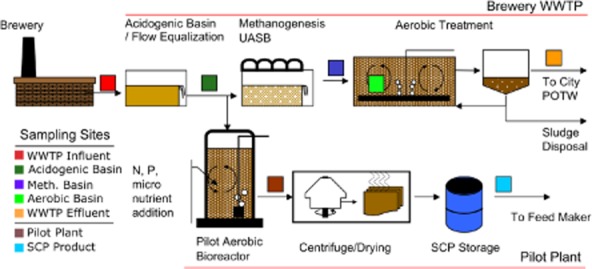
Overview of the study site showing the brewery treatment works (top) and the pilot-scale reactor (bottom). Coloured squares indicate sampling location and are used to illustrate figures throughout this paper.

**Fig 2:**
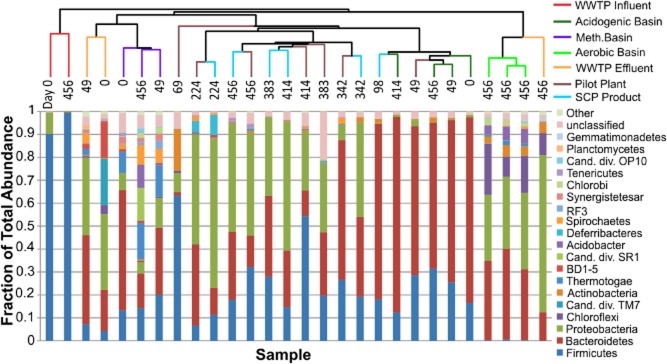
Phylum-level distribution shown sorted by UPGMA clustering of UniFrac unweighted distance. Clades common to a unit process colored based on Fig. 1. Stacked bar graphs of phyla distributions are sorted by total dataset rank abundance starting with the most common from the bottom. Leaves are labeled with the day of sampling from initial date.

### Tracking microbial response in the pilot bioreactor by principal component analysis

**Fig 3:**
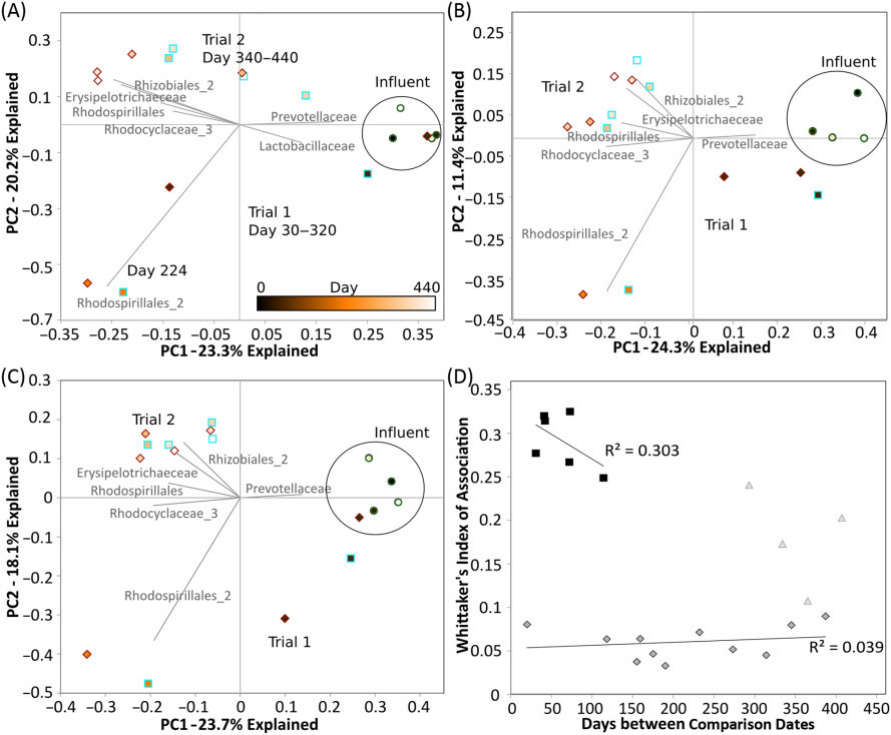
Microbial turnover across a bioreactor over two production trials. PCoA plots of (A) Whittaker's index of association, (B) unweighted UniFrac, (C) Sørensen index and (D) distance–decay of Whittaker's index of association. (A, B, C) PCoA plots include influent samples as a baseline (green circle) and pilot plant mixed liquor (brown diamond) and SCP (blue square) time courses (fill color indicates time progression). PCoA bi-plots of the level 4 SILVA 104 non-redundant database taxonomy of the most abundant OTUs are denoted. (D) Distance–decay pairwise comparisons are graphed according to the difference in days of time points. Distance–decay trends are seen only for sample comparisons for trial 2 (square) but not trial 1 (triangle), and all other comparisons (diamond).

When tracked over time, each trial run within the pilot plant produced the same pattern of shifts with the three beta diversity metrics studied (Fig. 3A–C). In trial 1, the pilot reactor community was initially similar to the influent environment and then shifted to a new community composition. Before trial 2, the reactor was drained and refilled but not re-inoculated or sterilized. In trial 2, a second microbial community developed that was different from the influent and trial 1. For principal component analyses (PCoAs) of the pilot bioreactor and influent, results did not change with jackknifing to 800 sequences per sample (Supporting Information Fig. S2). No significant time–decay relationship (representing steady succession or turnover) was seen in the study, and most comparisons between time points showed the same level of dissimilarity distance (Fig. 3D). Bi-plots of the most common family-level classifications of OTUs found in this study were graphed in conjunction with PCoA data (Fig. 3A–C) and showed a correlation of *Rhodospirillales* with trial 1, several family types from *Proteobacteria* with trial 2 and *Prevotellaceae* with the influent.

### Co-abundance, metastats and microbial lifestyle analysis of pilot bioreactor and SCP product OTUs

**Fig 4:**
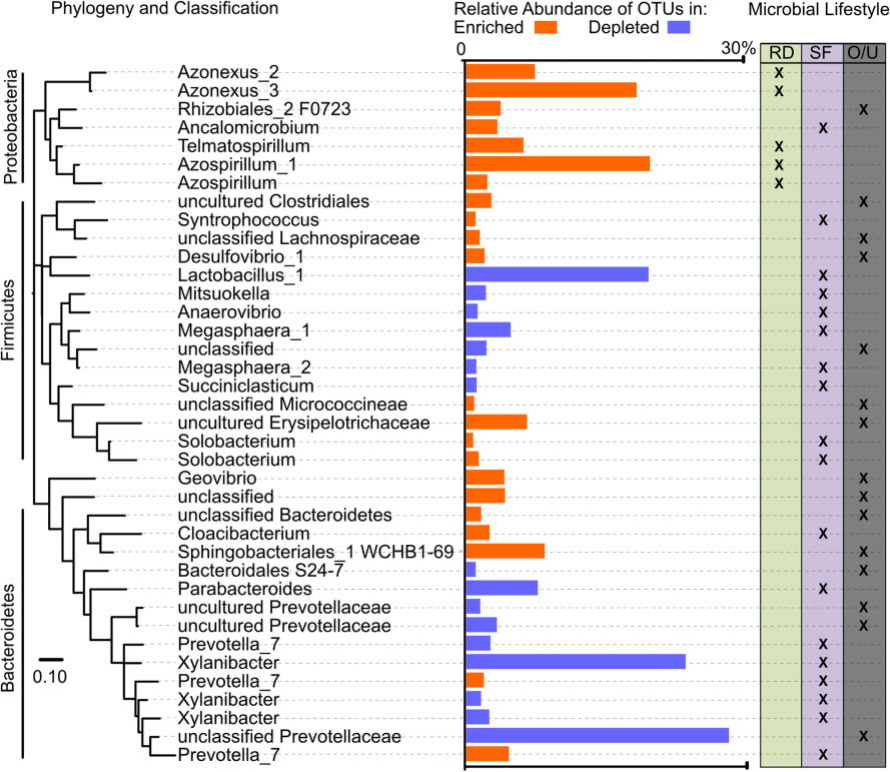
Phylogenetic tree and sequence abundance of depleted and enriched OTUs. Enriched OTUs (orange) and depleted OTUs (blue) show clustering by phylogeny and have differential metabolic roles when classified by rhizospheric diazotroph (RD, pea green), saccharolytic fermenter (SF, light purple) and other/unclassified (O/U, grey). References for metabolic assignment and phylogenetic tree bootstrap values in Supporting Information Fig. S6.

To address the question of which OTUs might be biotechnologically relevant, the variations of OTU abundances in the pilot reactor and SCP product samples over time were used to generate Bray–Curtis distances (a generalized distance metric) between repeatedly occurring OTUs. These data were clustered using UPGMA (Supporting Information Fig. S3) and identified that a small number of recurrent OTUs with high abundance (6.2% of OTUs and 67.6% of sequences) contributed to a deep clade within the clustergram. For these OTUs to be responsible for SCP formation, they should have a distribution that was enriched within the pilot bioreactor and SCP product when compared with the community of the acidogenic basin (which served as the influent community to the pilot bioreactor). When the MetaStats statistic was used to compare these OTUs with the pilot bioreactor influent distribution of OTUs, about 44.7% of the sequences (from the combined set of SCP and pilot bioreactor OTUs) were significantly enriched over the influent, and 29.6% of the sequences from the combined set were from OTUs that were significantly depleted compared with the influent (Supporting Information Fig. S4). When a phylogenetic tree was constructed from representative pyrotags of these OTUs, a phylogenetically coherent trend was observed. As shown in Fig. 4, when sequences from enriched or depleted OTUs were cross-referenced with the known microbial lifestyle of the taxonomic classification of the sequence, the depleted OTUs tended to be more from saccharolytic fermenters, primarily *Prevotellaceae*. Enriched OTUs tended to be from genera consisting of rhizospheric diazotrophs such as *Azospirillum*, *Azonexus* and *Telmatospirillum*, and a smaller fraction of saccharolytic fermenters. OTUs identified as having a rhizospheric diazotroph lifestyle were absent from both the pilot bioreactor influent and from depleted OTUs, but accounted for 39.4% of sequences from enriched OTUs in the pilot bioreactor and 73.0% of sequences from enriched OTUs in the final product.

### Full-length 16S sequences of rhizospheric diazotrophs from the pilot bioreactor

**Fig 5:**
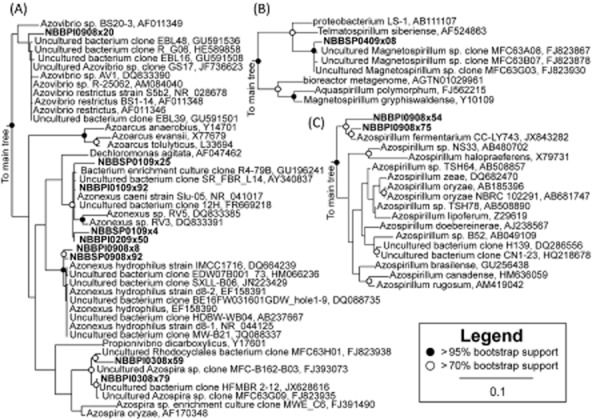
Phylogenetic diversity of full-length Sanger sequences related to rhizospheric diazotrophs detected from the pilot bioreactor and final product for sequences from (A) *Azoarcus*, *Azonexus* and *Azospira* clades, (B) *Magnetospirillum* and *Telmatospirillum* clades, (C) and *Azospirillum* clades. Symbols denoting bootstrap support values are for maximum likelihood analyses. Representative sequences for OTUs identified from this study are in bold.

Phylogenetic trees of full-length Sanger 16S sequences from rhizospheric diazotrophs in *Alphaproteobacteria* and *Betaproteobacteria* are shown in Fig. 5 (full phylogenetic tree in Supporting Information Fig. S5). In the *Betaproteobacteria* (Fig. 5A), near full-length sequences were related to known isolates of *Azospira* sp., *Azovibrio restrictus* and *Azonexus caeni* (but not genera *Azoarcus*). In the *Alphaproteobacteria* (Fig. 5B and C), near full-length clone sequences were not related to any of the known rhizospheric strains for *Magnetospirillum* and were related to one strain of *Azospirillum* [*Azospirillum fermentarium* CC-LY743 isolated from a fermentation tank (Lin *et al*., 2013)]. Closest basic local assignment search tool (BLAST) matches were commonly from strains isolated in large part from either wastewater and microbial fuel cell sources (Kaksonen *et al*., 2004; Quan *et al*., 2006; Borole *et al*., 2009; de Cárcer *et al*., 2011; Croese *et al*., 2011; Sun *et al*., 2011), as well as rhizosphere studies (Ashida *et al*., 2010; Knief *et al*., 2012) for which rhizospheric diazotrophs are primarily associated.

## Discussion

### Insights into microbial diversity in WWTPs

Prior to this work, it was unclear if large segments of the microbial community would pass through the entire treatment plant or if communities would shift entirely from one treatment regime to another. This study reveals that the influent waste beer from brewery process water was limited in bacterial diversity and had little impact on the colonization of subsequent unit processes in a brewery wastewater treatment works, and that the microbial community of each unit process remained largely self-selective. In concurrence with previous work on wastewater microbial variation (Fernandez *et al*., 2000; Wells *et al*., 2011; Werner *et al*., 2011), constant turnover of even the most common species was a normal occurrence in samples from this study. In terms of novel diversity discovery, unclassifiable OTUs were found in all environments downstream of the acidogenic basin. Particularly surprising was the number of unclassified OTUs found in the methanogenesis UASB and the clarifier wet-well outfall (purple and orange squares, Fig. 1). The design of the UASB may be one contributor to the diversity, as the sludge granules are known to be highly complex physical structures with differing chemical and microbial composition based on depth from the surface of the granule (Sekiguchi *et al*., 1999; Liu *et al*., 2002; Diaz *et al*., 2006). The clarifier wet well receives low settling COD effluent from clarifier operations prior to discharge from the plant and may be an overlooked environment for sampling, likely harbouring organisms related to wastewater treatment washout.

### Organisms responsible for SCP cell growth

The significantly depleted OTUs were found to be primarily saccharolytic fermentative anaerobes such as *Prevotella* (Fig. 4), whereas several separate types of diazotrophs (as well as some fermentative anaerobes) comprised much of the enriched sequences in the pilot bioreactor and SCP product. The presence of saccharolytic bacteria in aerobic conditions was unusual as *Prevotella* are strict anaerobes. In the final SCP product, even higher abundances of rhizospheric diazotroph sequences were seen than in the pilot bioreactor. We interpret the reduction of *Prevotella* and an increase in rhizospheric diazotrophs in SCP as indicating that the primary treatment effect responsible for SCP production was from rhizospheric diazotrophs and that the saccharolytic fermenter sequences, although common, may be from inactive cells washed in from the acidogenic basin. While diazotrophy exists across numerous phyla in nature, in this pilot bioreactor only diazotrophs from the *Proteobacteria* commonly associated with wastewater consortia were detected. The large relative abundance of diazotrophs seen in this study has a parallel in the treatment of certain high-strength wastewaters such as olive oil wastes and paper and pulp mill wastes where *Proteobacteria* rhizospheric diazotrophs were isolated and *nifH* genes and transcripts and nitrogenase activity detected (Papadelli *et al*., 1996; Clark *et al*., 1997; Gauthier *et al*., 2000; Bowers *et al*., 2008). These environments are often replete with simple carbon sources, yet are relatively limited in nitrogen and oxygen and require a diverse suite of microorganisms to generate fixed nitrogen to support the broader microbial consortia. In the pilot reactor of our studied system, aeration estimates produced DO levels 0.1–0.5 mg l^−1^ in the pilot reactor. There may be similarities with this environment and the rhizospheric habitat where such organisms autochthonously exist, particularly the abundance of simple organic acids from acidogenesis, which in the rhizosphere are released from plant roots to diazotrophs (Christiansen-Weniger *et al*., 1992).

However, we must caution that without further investigation, nitrogen fixation cannot conclusively be attributed as the primary biological advantage, especially since unlike conventional brewery wastewater treatment, nitrogen (as urea) was added to excess, and nitrogen fixation would be rapidly suppressed by the presence of fixed nitrogen. A major challenge in this study was monitoring the entire mixed liquor of such large bioreactors; therefore, it is possible that urea was not optimally dissolved, mixed or sorbed to microbial flocs. Another possible association is that the saccharolytic fermenting anaerobic bacteria in the acidogenic upstream treatment produced easily accessible organic acids that become the influent of the pilot bioreactor, where microbes adapted to have affinity for organic acids, and other pilot bioreactor conditions are then enriched. Under both scenarios, the wide presence of wastewater-specific diazotrophs found in this study indicates a potential target for future metabolic biotechnology exploitation. For example, because of the variable flow nature of batch fermentation in beer production, breweries have large flow equalization basins to regulate the supply of wastewater to continuous flow treatment stages. The conversion of such basins to an intentional acidogenic pretreatment stage upstream of the treatment bioreactors is one possibility to produce a constant supply of organic acids for uptake and conversion to SCP by aerobic or microaerophilic organic acid consuming microbes. Another potential avenue of research centres on the use of methanogenic UASBs as an inoculant source for these types of diazotrophs to seed their growth.

In summary, this study highlights the overall distinctiveness of each treatment stage within a single process WWTP, especially the occurrence of *Prevotella* and related saccharolytic fermenters in flow equalization basins and the role of wastewater-associated rhizospheric diazotrophs in high-strength wastewaters. This research indicated that rather than growing a single culture of these organisms, endogenous enrichment of rhizospheric diazotrophic bacteria in high-strength wastewater treatment systems can be used to produce SCP and should be studied as a way to deliberately manipulate microbial ecology for the production of a high protein content ingredient for aquaculture feed. However, before large-scale implementation of this technology can occur, several challenges remain to bring such a product to market such as regulatory and feed safety approval, scale up of production and large-scale animal feeding trials.

## Experimental procedures

### Project site description and sample handling

Figure 1 shows the brewery WWTP and pilot reactor research site used in this study (New Belgium Brewing, Fort Collins, CO, USA). The brewery treatment works itself consists of an unaerated influent flow equalization basin with acidogenic conditions, followed in series by a methanogenesis basin consisting of an UASB bioreactor. Next follows an aerobic treatment basin and clarifier for activated sludge growth and settling prior to discharge to the city publicly owned treatment works.

The pilot reactor received wastewater from the acidogenic basin to aerobically treat the wastewater at low mean cell residence time (< 8 days) with nutrient addition (N as urea, P as phosphoric acid and a customized micronutrient cocktail) to increase the protein concentration via growth of cell mass. The pilot bioreactor was initially fed and seeded from the methanogenesis UASB for about 1 month prior to the start of trial 1 before being switched to the acidogenic basin.

Two separate production trials using the pilot bioreactor were completed over a 450 day study period (trial 1: day 30–320, trial 2: day 340–440), and enough SCP was consistently produced at > 55% crude protein content for use in commercial feeding trials (Logan *et al*., 2011). Sampling locations and reactor conditions are shown in Fig. 1 (colored squares) and Table 1. Treatment plant influent samples (Fig. 1, red square) were taken from the waste influent receiving line upstream of the acidogenic basin. Acidogenic basin (dark green square) samples were taken from basin mixed liquor. Methanogenesis UASB (purple square) samples were taken from the basin outfall stream. Aerobic basin (light green square) samples were taken from basins directly, and clarifier wet well (orange square) was taken from the wet-well outfall channel. Pilot bioreactor samples (brown square) were taken from an effluent sampling port leading to the drying and centrifuge assembly. Finally, dried SCP product (blue square) was sampled directly from storage containers containing the most recent batch of manufacture. Liquid grab samples from basins and outfalls were collected over the study period in autoclaved 1-L serum bottles (rinsed with sample first) and returned to the lab and spun down at 10 000 relative centrifugal force (RCF) for 5 min. Grab samples and dry SCP product samples were stored at −20°C until DNA extraction with a Mo-Bio PowerSoil Kit (Carlsbad, CA, USA) per the manufacture's protocol with the exception of a 1 min bead beating step in a Biospec (Bartlesville, OK, USA) MiniBeadbeater-8 instead of vortex bead beating.

### Wastewater chemical analysis

WWTP characterization (N, P, COD and VFA) analysis was conducted using commercial Hach TNT kits (Loveland, CO, USA). TSS was measured using filter paper and drying at 105°C (APHA, 1985) collected daily from January to March 2009. Pilot bioreactor MLSS (Royce 711, Australia) measurements and pH were collected daily from October 2008 to May 2009. Grab samples collected at the same time as nucleic acid samples were returned to the lab for organic acid analysis. An ion exclusion organic acid column Animex HPX-87H (Bio-Rad, Hercules, CA, USA) with a pre-column filter (Bio-Rad) was used with an Agilent 1100 HPLC. Samples were spun (10 000 RCF, 5 min) and filtered (0.45 μm) before use. Standards for formic, acetic, lactic, propionic, succinic, butyric, isobutyric, valeric, isovaleric, 2-methylbutyric and citric acid were run at three intervals in replicates of three with an injection volume of 50 um. Running buffer consisted of 0.04 N phosphoric acid, 0.60 ml min^−1^, 40°C, 35 min. Diode array detector signal frequency was 210 nm, reference 360 nm. Concentrations of all detected organic acids were summed to determine total organic acid content in samples.

### Sampling and Sanger sequencing

Samples were collected over a period of 1 year from throughout the brewery treatment works as well as from the pilot plant and final dried product to track changes in community composition across treatment stages and in time. Sample DNA extraction, Sanger sequencing and full-length 16S small subunit (SSU) ribosomal RNA (rRNA) gene bioinformatic methods were adapted from Sahl and colleagues (2010) for 8F and 1492R bacterial primers and with details listed in the Supplemental Methods. Briefly, DNA was extracted from samples and amplified by polymerase chain reaction (PCR) followed by vector cloning of amplicons and Sanger sequencing of the T3/T7/515F reads, and finally contig formation to determine full-length 16S SSU rRNA gene reads. Reads were clustered at the 97% OTU level and unique representative sequences extracted. Representative sequences derived from Sanger sequencing OTUs of full-length 16S sequence clones from *Proteobacteria* were used with 64 full-length 16S sequences of BLAST closest matches and 37 SILVA reference sequences for phylogenetic reconstruction. These sequences were trimmed to a contiguous aligned [Nearest Alignment Space Termination (NAST)-based aligner of mothur (Schloss, 2009)] 1142 bp length and used with the pos_var_bac ‘0’ filter in phyml (Guindon *et al*., 2010) with default parameters and 100 bootstraps for detailed phylogenetic comparison.

### Pyrosequencing and bioinformatics pipeline

Pyrosequencing PCR of the 16S SSU rRNA gene using the bacterial 27F and 338R primers with sequence barcoding (Hamady *et al*., 2008) adapted from Sahl and colleagues (2010) and processed using qiime (Caporaso *et al*., 2010), with details listed in the Supplemental Methods. Briefly, sequences were denoised filtered, and clustered using qiime, then aligned and classified using the NAST-based aligner and classifier of mothur (Schloss, 2009) trained on the customized SILVA 104 non-redundant database (80% confidence level cut-off). Note that taxonomic assignments in figures and text in this paper with underscores or number assignments at the end (e.g. firmicutes_bacilli or azonexus_2) indicate paraphyletic classification in the SILVA reference tree system.

### Alpha and beta diversity analysis

qiime was used to compute alpha diversity estimates of the Chao1 and ACE metrics (Colwell, 2009). The CatchAll statistic (Bunge, 2011) was also used with default parameters, and the best model total species observed values were used. All alpha diversity estimators were computed with samples rarified up to the same sequencing depth with 50 replicates each. Coverage estimates were based on the percentage of observed OTUs to the Chao1 estimator as a low estimate and to the CatchAll as the high estimate using all sequences per sample for computation. Several metrics of beta diversity originally derived from classical macroecology [particularly plant communities (Sørensen, 1948; Whittaker, 1972)], such as the Sørensen (a qualitative sharing metric) and Whittaker's Index of Association (quantitative relative abundance sharing metric), were chosen to study the pilot plant system. These have been applied for the study of variations in bacterioplankton communities across distance in the North Pacific (Hewson *et al*., 2006) and could indicate temporal-spatial distance–decay relationships. Additionally, based on recent work on wastewater variation (Zhang *et al*., 2012), the unweighted UniFrac metric (Lozupone *et al*., 2011) was used to compare wastewater samples. qiime was used to calculate the beta diversity metrics of unweighted UniFrac (using a whole tree of the entire dataset), the complement of Whittaker's index of association (Legendre and Legendre, 1998) (using a custom python script rather than the qualitative Whittaker's found in qiime), and the qualitative Sørensen index of similarity. UPGMA clusters and principal coordinate analysis were determined using qiime scripts. Distance matrices from each metric were also jackknife subsampled to the smallest sequence count to examine the sensitivity of sequencing depth on clustering and principal coordinate analysis. Bi-plots of only the top 10 most commonly found taxonomic families were displayed.

### Determining biotechnologically relevant OTUs

While it is useful to compare environments over time in a holistic manner with beta diversity metric analysis, we also wanted to identify the distribution of OTUs responsible for the community shift, especially the fraction that indicates SCP formation. To determine organisms that might be of biotechnological interest, two tests were used. The first was to test if OTUs are co-abundant (i.e. co-incident in time and relative abundance). This can highlight patterns of variation of consortia in response to natural wastewater source variations. Additionally, a significance test was used to determine OTUs that were differentially enriched in the mixed liquor as compared with the reactor influent.

To understand the co-abundance of OTUs in both the pilot plant and final SCP product, OTUs that were present in these two environments were used. OTUs were included if an OTU was present in these two environments (pilot bioreactor and final product) more than once and had more than two sequences associated with it. 319 out of 1604 OTUs passed this criteria. The Bray–Curtis pairwise distance metric (Legendre and Legendre, 1998) was computed for each OTU based on the abundances of each OTU across SCP and pilot bioreactor samples (*n* = 13).

Each OTU was then grouped into a functional category based on the metabolic profile of the nearest taxonomic representative found in Bergey's Index (Brenner *et al*., 2011) (characteristic of the entire known genus or family level) or related primary literature (of the genus level only). A conservative approach was taken where clades with significant known metabolic variation or poor classification (at the genus level) were labeled as ‘unknown or unclassified’.

OTUs were additionally compared by online metastats (http://metastats.cbcb.umd.edu/detection.html) (White *et al*., 2009), a program developed to examine differential abundance of elements associated between patients from a control and treatment category for medical microbiome studies. Abundance information from the series of acidogenic basin samples (representing the influent to the pilot plant) and the pilot plant mixed liquor samples were used as the pre- and post-treatment cases with a threshold *p*-value < 0.05 and a difference in average relative abundance of ± 1% absolute magnitude as being significantly ‘enriched’ or ‘depleted’ respectively.

A major concern in this work was the role of PCR and extraction biases in calculating co-abundance dissimilarity distances. This work did not seek and does not serve to capture the true compositional nature of the sample, but rather relative abundance data is used to understand patterns in OTU distributions undergoing the same DNA extraction, amplification and analysis conditions. Another concern was misidentification or the inclusion of spurious sequences leading to poor taxonomic identification. Therefore, no conclusions are made on any single OTU, and full-length 16S SSU Sanger-based gene sequencing was used to verify the findings of short sequencing reads.

### Nucleotide accession numbers and online resources

A total of 809 Sanger sequences were deposited in GenBank under accession numbers JQ072092–JQ072899. Sanger sequences were named according to the location and date in which they were sampled [i.e. NBBEQMMYY_44; where NBB = New Belgium Brewing, EQ = flow equalization/acidogenic basin, ME = methanogenic UASB, PI = pilot plant, SP = SCP product, AB = aerobic basin, OT = wet well outfall, MMYY = (month/year) 44 = sequence number]. 454 pyrotags taken from quality score processing were submitted in FASTQ format to mg-rast (ID 4477674.3). Custom python scripts and interactive Krona pie charts (Ondov *et al*., 2011) used in this study can be obtained from http://inside.mines.edu/~jspear/resources.html.

## Supporting Information

**Fig. S1.** Jackknife UPGMA node sensitivity analysis for Figure 2. **Fig. S2.** Jackknife PCOA ellipsoid plots, subsampled to 800 sequences per sample with 100 jackknife replicates. **Fig. S3.** OTUs in a pilot reactor and SCP product clustered over time. **Fig. S4.** Breakdown of sequences in co-abundance and lifestyle analysis showing the distribution of enriched and depleted sequences across pilot bioreactor mixed liquor and final SCP product. **Fig. S5.** Phylogenetic diversity of full-length Sanger sequences related to rhizospheric diazotrophs detected from the pilot bioreactor and final product. **Fig. S6.** Phylogenetic tree and sequence abundance of depleted and enriched OTUs.
