# Supplementary material for: Microbial response to single-cell protein production and brewery wastewater treatment
Source: Microb Biotechnol. 2014 May 16;8(1):65–76. doi: 10.1111/1751-7915.12128 (PMC4321374; doi:10.1111/1751-7915.12128)
Supplement: Supplementary file 1 [file mbt20008-0065-sd1.zip › Supplemental Information.pdf]

Supplemental Information for:

Microbial Response to Single Cell Protein Production and Brewery Wastewater Treatment

Running Title: Microbial Diversity in Single Cell Protein

Authors: Jackson Z. Lee[1], Andrew Logan[2], Seth Terry[2] and John R. Spear[1#]

[1]Department of Civil and Environmental Engineering, Colorado School of Mines, Golden, CO 80401

[2]Oberon FMR, Inc., Aurora, CO 80045

[#]Corresponding author, jspear@mines.edu, Phone: +1 (303) 273-3497
Fax: +1 (303) 273-3413

Author Address Change:
Jackson Z. Lee, NASA Ames Research Center, MS 239-4, Moffett Field, CA 94035

Supplemental Methods

Sanger Sequencing

Samples were collected over a period of 1 year from throughout the brewery treatment works as well as from the pilot plant and final dried single cell protein (SCP) product to track changes in community composition across both treatment stages and in time. Sample DNA extraction, Sanger sequencing, and full-length 16S small sub-unit (SSU) rRNA gene analytic and bioinformatic methods were adapted from Sahl, et al. (2010) for 8F and 1492R bacterial primers. In brief, template DNA was amplified with the 8F (5'-AGAGTTTGATCCTGGCTCAG-3') (Lane, 1991) and 1492R (5' – ACCTTGTTACGACTT – 3') (Lane, 1991) primers using Promega PCR Master Mix (Promega Corp., Madison, WI), using the thermal-cycler program 94°C, 2:00 min initial denaturation, 30 X at 94°C 30 sec denaturation, 55.5°C, 1:00 min annealing, 72°C 1:30 min extension, 72°C, 12:00 min final extension and 4°C final hold. PCR product was then Montage gel purified (Millipore, Billerica, MA) and cloned into Invitrogen (Life Technologies, Grand Island, NY) Top10 electro-competent cells. Clone plates of 96 wells were Sanger sequenced (3 reads; T3, T7, 515F) on a MegaBACE 1000 dye-terminating sequencer. Chromatograms were called using Phred and Phrap (Ewing et al., 1998) and quality controlled (Q30) and joined into contigs by XplorSeq (Frank, 2008). Full-length chimera-checked (Mallard) (Ashelford et al., 2006) 16S rRNA gene sequences were then clustered and aligned using the NAST-based aligner in mothur (Schloss, 2009) using the Silva Non-Redundant 104 database (Pruesse et al., 2007) and incorporated into the ARB (Ludwig et al., 2004) reference tree alignment of the database for "Silva" (ENA-EBI curated) with taxonomy determination by parsimony insertion. Nearest neighbor Silva taxonomies were copied onto the novel sequences in the "silva_tax" field. A subset of the bacterial domain in the Silva 104 Non-Redundant sequences (pintail_slv > 90, seq_quality_slv > 50, align_quality_slv > 50, ambig_slv = 0) with the

augmented Sanger sequences was then exported for downstream bioinformatics processing. Sequences were truncated from *E. coli* alignment position 100 to 8099 and were pruned of sequences less than 300 bp in length by a custom python script. A filtered alignment file composed of the pos_var_bac Silva mask at threshold '0' was created for tree construction also by a custom python script. These sequences were then used as the Silva Non-Redundant 104 customized database used for downstream analysis.

Pyrosequencing & Bioinformatics Pipeline

Pyrosequencing PCR of the 16S SSU rRNA gene using the bacterial 27F and 338R primers (corresponding to the V1-V2 variable regions of the 16S rRNA gene) with sequence barcoding (Hamady et al., 2008) was adapted from Sahl et al. (2010). Quantitative PCR (qPCR) cycling was completed using Promega Mastermix and SybrGreen and stopped at peak fluorescence (monitored on a LightCycler, Roche) using the thermal-cycler program 94°C, 2:00 min initial denaturation, 18-24 X at 94°C 30 sec denaturation, 52°C, 20 sec annealing, 72°C 1:00 min extension. qPCR product was quantified on a gel using a Bioanalyzer and pooled in equal proportion. The final pool was purified with an E.N.Z.A. gel extraction kit (Omega, Norcross, GA). Sequencing was completed at EnGenCore (Selah Genomics, South Carolina) on a 454 platform with multiplex amplicon barcoded sequencing. Using QIIME (Caporaso et al., 2010), raw sequencing files containing pyrosequences were denoised (default parameters) (Reeder and Knight, 2010) into flow-gram clusters and quality-controlled based on average quality score (>Q27), read length outlier screening, ambiguous bases (0) and homopolymer count (<10) (Huse et al., 2007). Operational Taxonomic Units (OTUs) of clustered sequences (UCLUST (Edgar, 2010), 97% similarity, default parameters) were filtered for chimeras using UCHIME (default parameters, de novo mode) (Edgar et al., 2011). The remaining OTUs were aligned using the NAST-based aligner and classifier of mothur (Schloss, 2009) trained on the customized Silva 104 non-redundant database.

Masked pos_var_bac ‘0’ aligned sequences were used with FastTree (Price et al., 2009) (default parameters) to generate a tree of all unique OTUs for use in Fast-UniFrac (Hamady et al., 2009) and phylogeny examination. The naïve Baysian classifier implementation in mothur (trained on the augmented Silva 104 database) was used to classify OTUs and determine taxonomy (80% confidence level cutoff). Note that taxonomic assignments in figures and text in this paper with underscores or number assignments at the end (e.g. firmicutes_bacilli or azonexus_2) indicate non-monophyletic classification in the Silva reference tree system.

References

Ashelford, K.E., Chuzhanova, N.A., Fry, J.C., Jones, A.J., and Weightman, A.J. (2006) New Screening Software Shows that Most Recent Large 16S rRNA Gene Clone Libraries Contain Chimeras. *Appl Environ Microbiol* **72**: 5734–5741.

Caporaso, J.G., Kuczynski, J., Stombaugh, J., Bittinger, K., Bushman, F.D., Costello, E.K., et al. (2010) QIIME allows analysis of high-throughput community sequencing data. *Nat Meth* **7**: 335–336.

Edgar, R.C., Haas, B.J., Clemente, J.C., Quince, C., Knight, R. (2011) UCHIME improves sensitivity and speed of chimera detection, *Bioinformatics*

Edgar, R. C. (2010) Search and clustering orders of magnitude faster than BLAST. *Bioinformatics*

Ewing, B., Hillier, L., Wendl, M.C., and Green, P. (1998) Base-Calling of Automated Sequencer Traces Using Phred. I. Accuracy Assessment. *Genome Research* **8**: 175–185.

Frank, D. (2008) XplorSeq: A software environment for integrated management and phylogenetic analysis of metagenomic sequence data. *BMC Bioinformatics* **9**: 420.

Hamady, M., Lozupone, C., and Knight, R. (2009) Fast UniFrac: facilitating high-throughput phylogenetic analyses of microbial communities including analysis of pyrosequencing and PhyloChip data. *ISME J.*

Hamady, M., Walker, J.J., Harris, J.K., Gold, N.J., and Knight, Rob (2008) Error-correcting barcoded primers for pyrosequencing hundreds of samples in multiplex. *Nat Meth* **5**: 235–237.

Huse, S., Huber, J., Morrison, H., Sogin, M., and Welch, D. (2007) Accuracy and quality of massively parallel DNA pyrosequencing. *Genome Biology* **8**: R143.

Lane, D.J. (1991) 16S/23S rRNA sequencing. In, Stackebrandt,E. and Goodfellow,,M. (eds), *Nucleic acid techniques in bacterial systematics*. Wiley, Chichester, UK, pp. 115–175.

Ludwig, W., Strunk, O., Westram, R., Richter, L., Meier, H., Yadhukumar, et al. (2004) ARB: a software environment for sequence data. *Nucleic Acids Res* **32**: 1363–1371.

Price, M.N., Dehal, P.S., and Arkin, A.P. (2009) FastTree: Computing Large Minimum Evolution Trees with Profiles instead of a Distance Matrix. *Mol Biol Evol* **26**: 1641–1650.

Pruesse, E., Quast, C., Knittel, K., Fuchs, B.M., Ludwig, W., Peplies, J., and Glockner, F.O. (2007) SILVA: a comprehensive online resource for quality checked and aligned ribosomal RNA sequence data compatible with ARB. *Nucl. Acids Res* **35**: 7188–7196.

Reeder, J. and Knight, R. (2010) Rapidly denoising pyrosequencing amplicon reads by exploiting rank-abundance distributions. *Nat Meth* **7**: 668–669.

Sahl, J.W., Fairfield, N., Harris, J.K., Wettergreen, D., Stone, W.C., and Spear, J.R. (2010) Novel microbial diversity retrieved by autonomous robotic exploration of the world's deepest vertical phreatic sinkhole. *Astrobiology* **10**: 201–213.

Schloss, P.D. (2009) A High-Throughput DNA Sequence Aligner for Microbial Ecology Studies. *PLoS ONE* **4**: e8230.

Schloss, P.D., Westcott, S.L., Ryabin, T., Hall, J.R., Hartmann, M., Hollister, E.B., et al. (2009) Introducing mothur: Open-Source, Platform-Independent, Community-Supported Software for Describing and Comparing Microbial Communities. *Appl Environ Microbiol* **75**: 7537–7541.

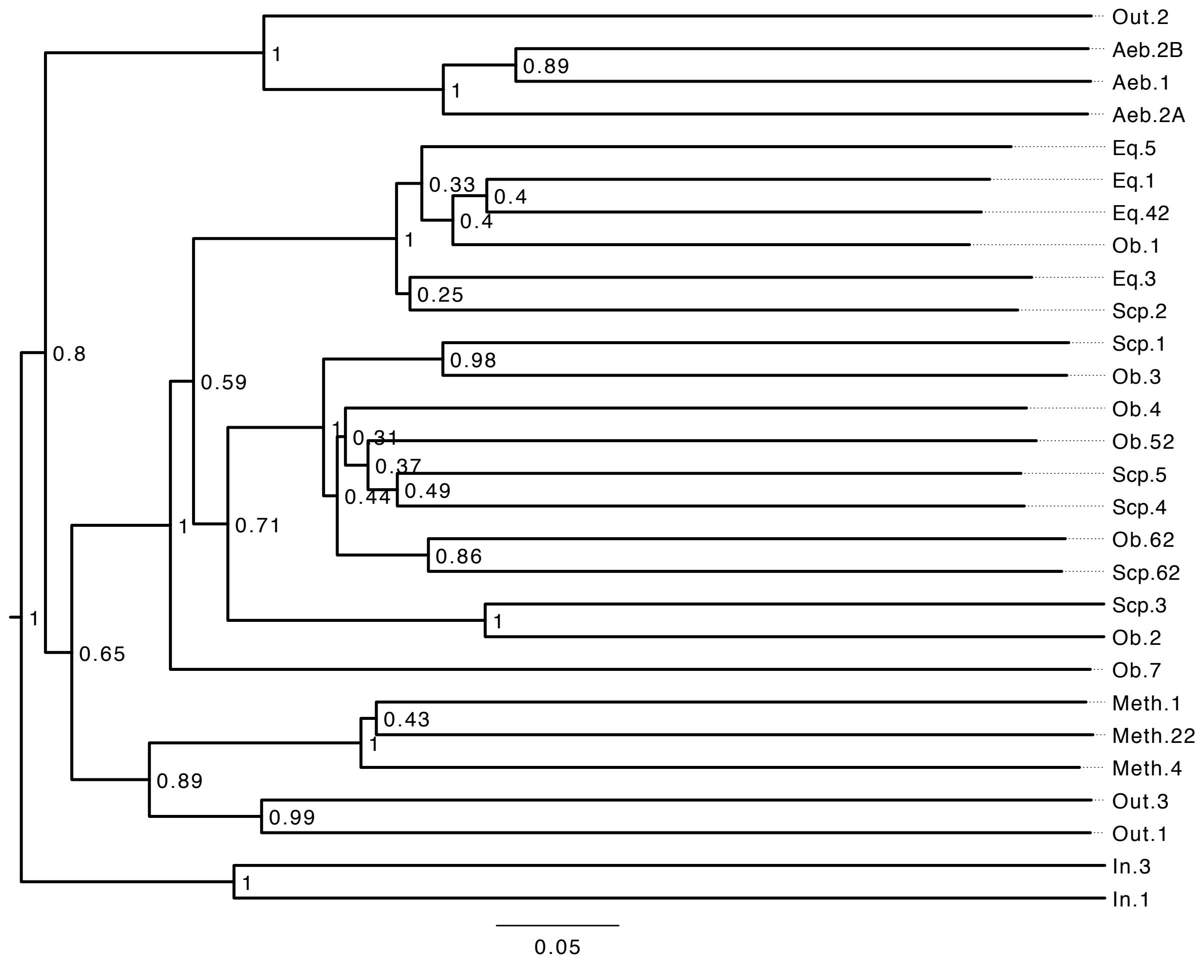

Figure S1. Jackknife UPGMA node sensitivity analysis for Figure 2. All samples were subsampled to 500 sequences per sample with 100 jackknife replicates. A consensus tree was selected and fraction of occurrence of a node computed. Out= clarifier outfall, Aeb= aerobic basin mixed liquor, Eq= acidogenic flow equalization basin mixed liquor, Ob=pilot bioreactor sample port, Scp= dried SCP product, Meth= methanogenesis UASB mixed liquor, In= WWTP influent sample port

Figure S2. Jackknife PCOA ellipsoid plots, subsampled to 800 sequences per sample with 100 jackknife replicates; Whittaker's (A); Unweighted Unifrac (B); and Sorensen (C).

(A)

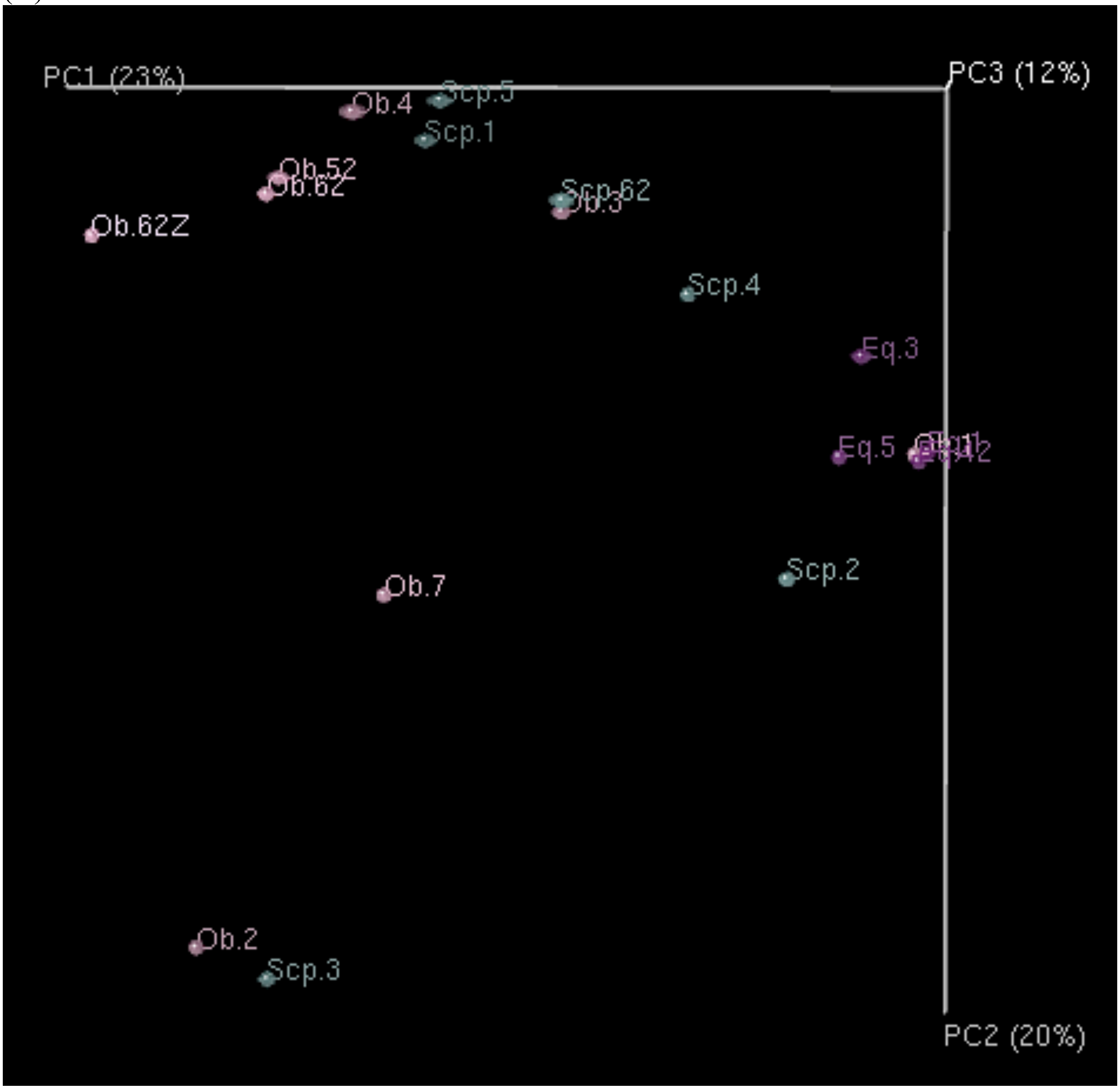

(B)

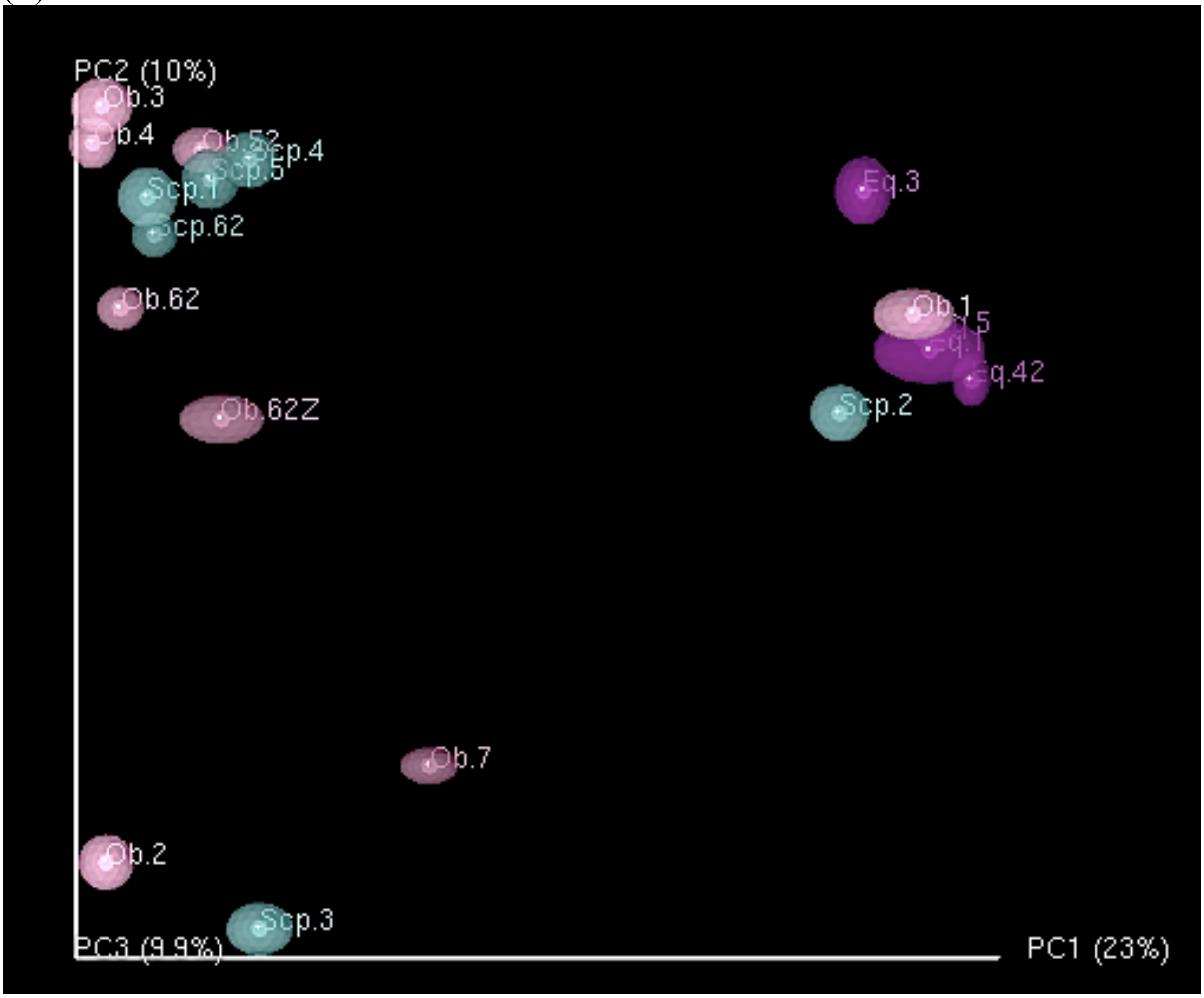
PC2 (10%)
Ob.3
Ob.4
Ob.52
Scp.4
Scp.5
Scp.1
Scp.62
Ob.62
Ob.62Z
Eq.3
Ob.1
Eq.5
Eq.1
Eq.42
Scp.2
Ob.7
Ob.2
Scp.3
PC3 (9.9%)
PC1 (23%)

(C)

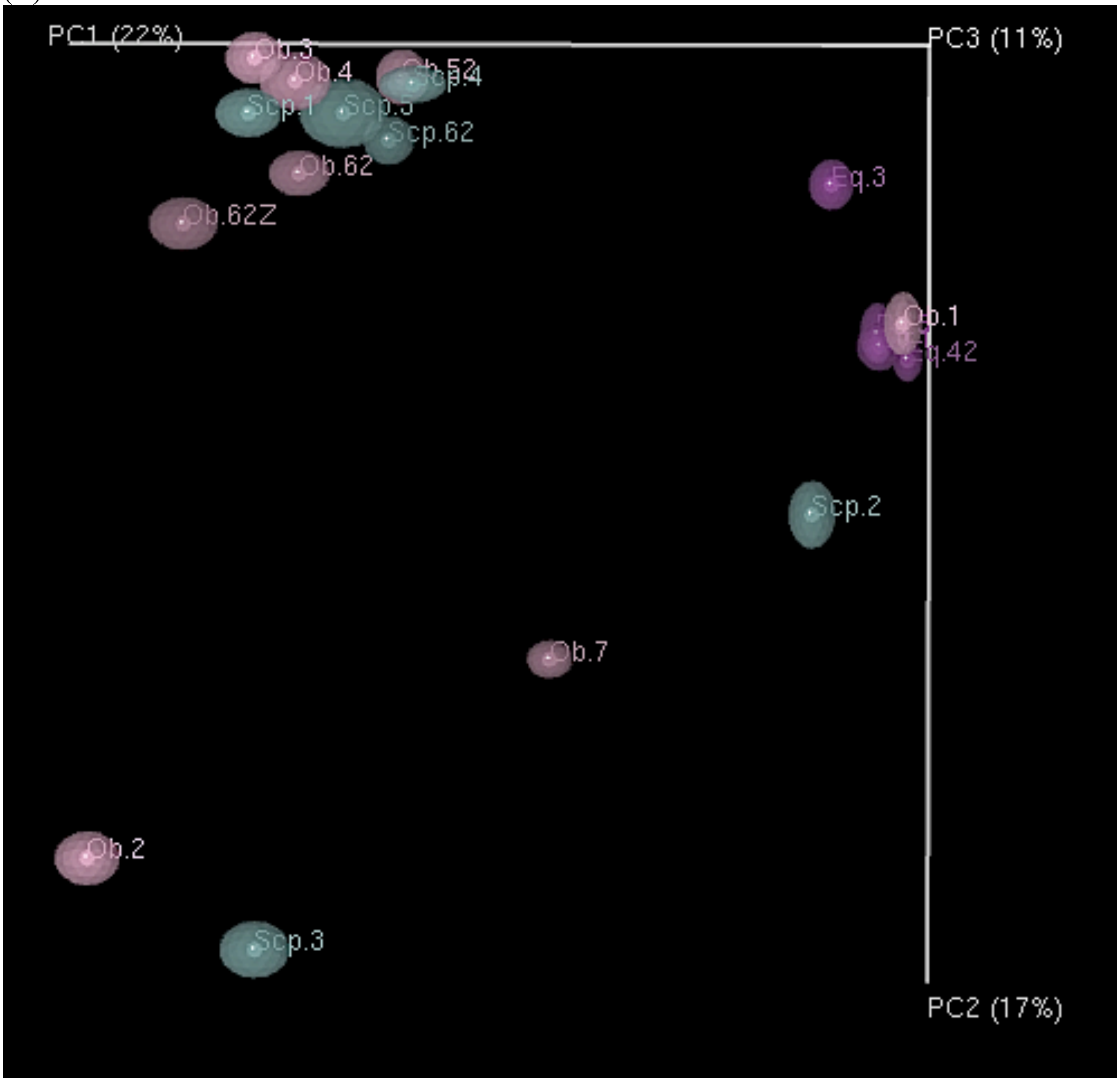

PC1 (22%)
PC3 (11%)
PC2 (17%)
Ob.3
Ob.4
Scp.1
Scp.62
Ob.62
Ob.62Z
Eq.3
Ob.1
Eq.42
Scp.2
Ob.7
Ob.2
Scp.3

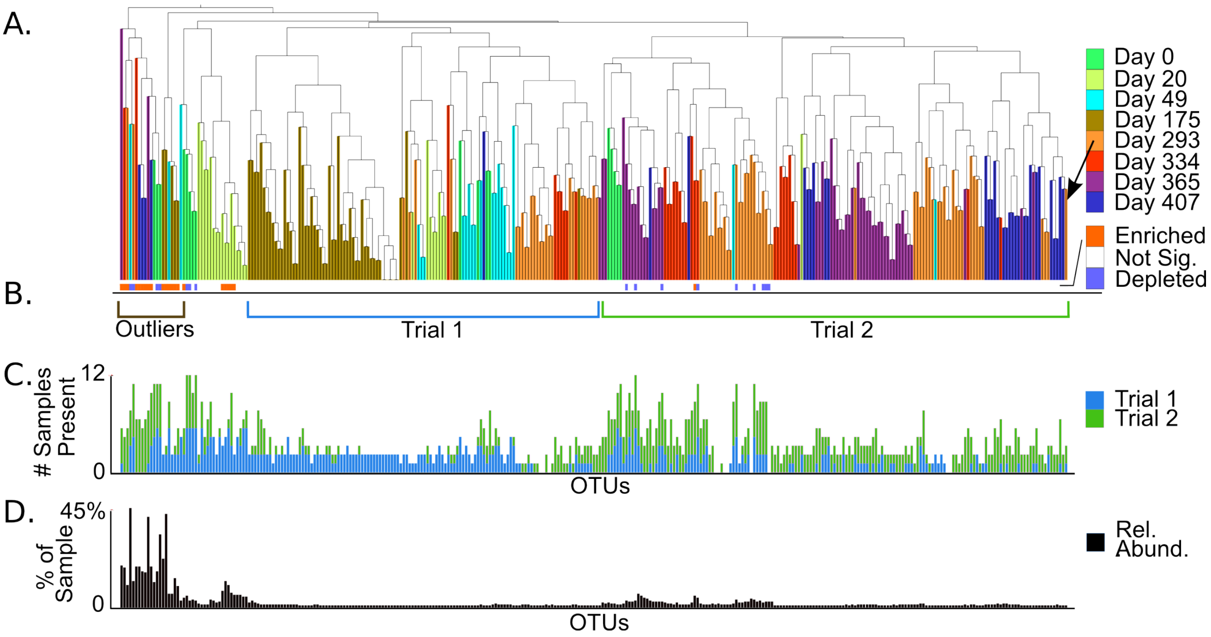

Figure S3. OTUs in a pilot reactor and SCP product clustered over time. OTUs were UPGMA clustered by co-abundance based on Bray-Curtis distance (A). Each leaf is labeled with the color of the date of its maximum abundance and shows that samples tended to cluster based on maximum abundance date at shallow levels in the clustergram.

Three deep clustergram associations were observed: deep clusters of outlier OTUs not associated with deep clades, OTUs occurring within a large clade associated with Trial 1, and OTUs occurring within a large clade associated with Trial 2. These are marked by the brown, light blue, and light green horizontal lines respectively.

Clades were marked if significantly enriched or depleted according to the MetaStats statistic (B). The distribution of OTUs during each trial is shown in (C) and show that the occurrence of OTUs in a trial was diagnostic for deep clusters shown in (B). The maximum abundance of each OTU is shown in (D) and showed that maximum abundance, regardless of date or trial occurrence, was responsible for the outlier cluster.

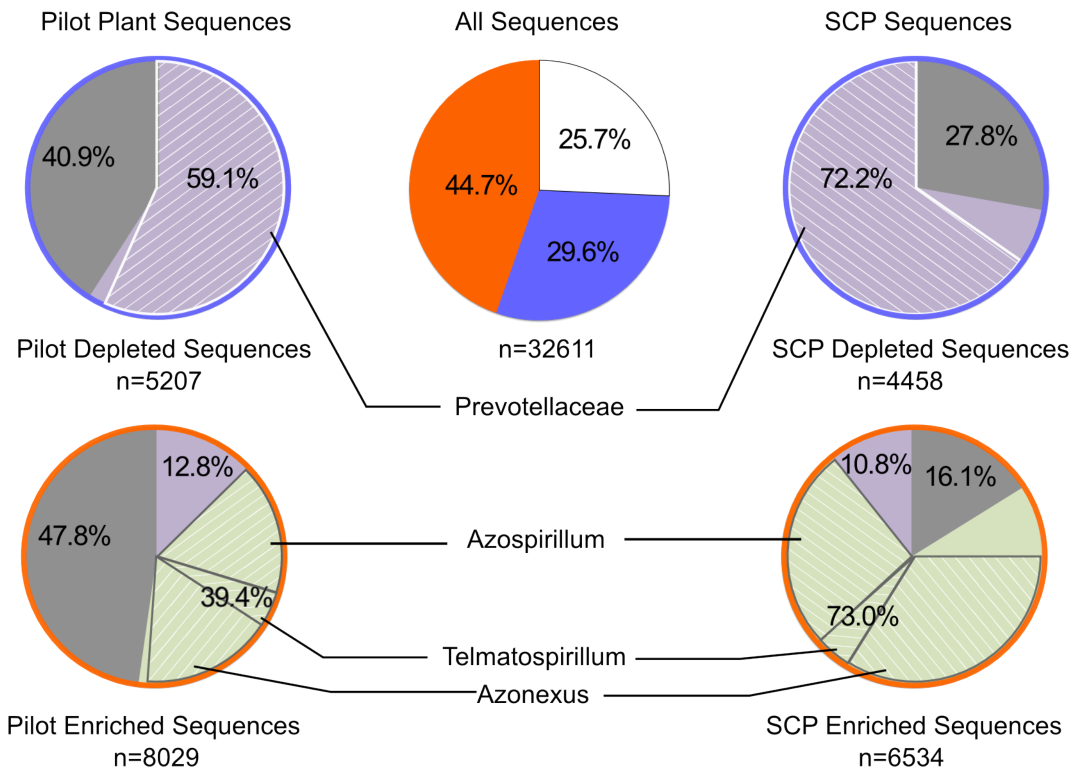

Figure S4. Breakdown of sequences in co-abundance and lifestyle analysis showing the distribution of enriched and depleted sequences across pilot bioreactor mixed liquor and final SCP product.

Enriched sequences are sequences from OTUs identified by Metastats as being significantly enriched over the influent (and respectively depleted sequences from significantly depleted OTUs) and are also from OTUs >1% average sample relative abundance. Enriched (orange), depleted (blue), and not significant (white) sequences are broken down into depleted and enriched categories (top and bottom respectively) and into pilot plant and SCP categories (left and right respectively).

Each subcategory has colors labeled based on metabolic lifestyle as from Figure 4 (purple, saccharolytic fermenters, pea green, rhizospheric diazotroph) and the percentage of sequences falling into that metabolic lifestyle is labeled in each wedge. In depleted categories, the family *Prevotellaceae* is broken out by a white striped wedge. In enriched categories, three different genera of rhizospheric diazotrophs are broken out by different white striped wedges.

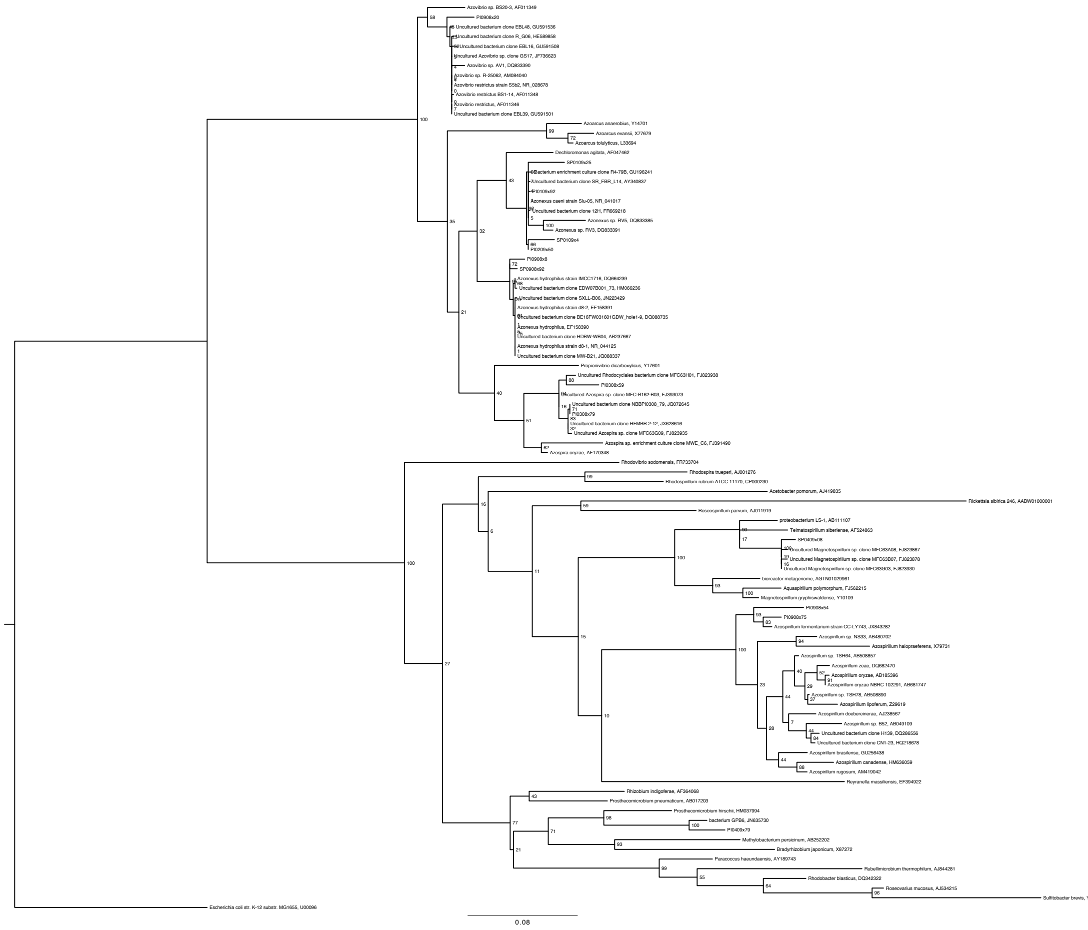

Figure S5. Phylogenetic diversity of full-length Sanger sequences related to rhizospheric diazotrophs detected from the pilot bioreactor and final product. E. coli U00096 was used as an outgroup. Node labels indicate bootstrap support values for ML analyses.

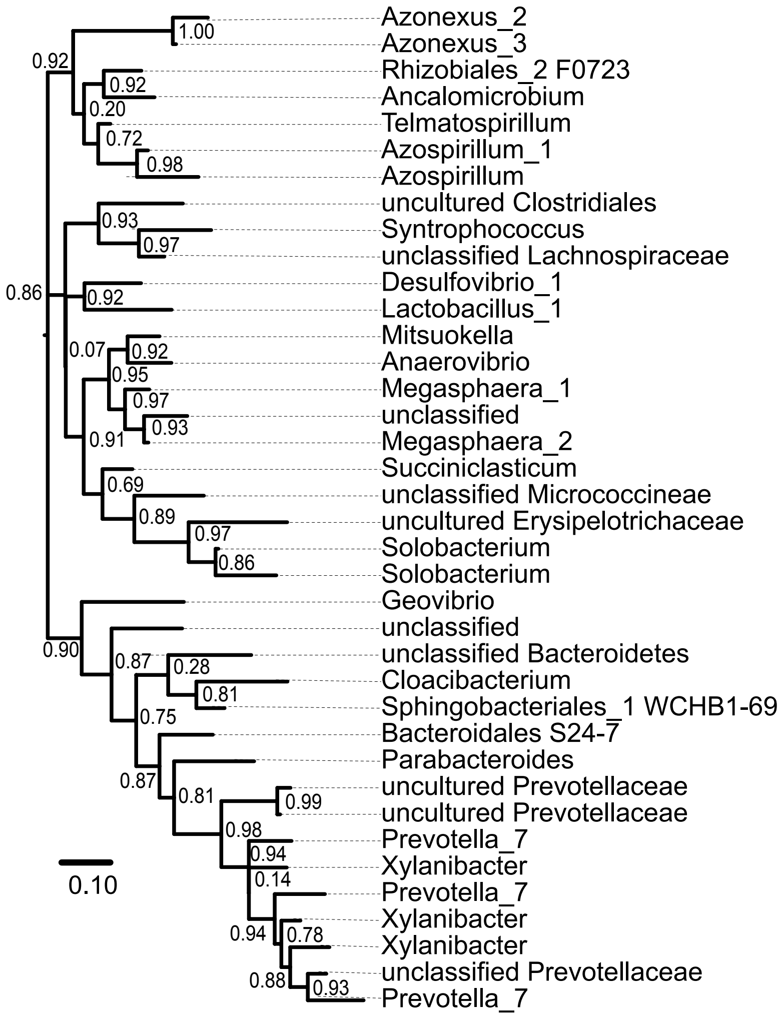

Figure S6. Phylogenetic tree and sequence abundance of depleted and enriched OTUs. FastTree with default values was used on pyrotag sequence results generated from custom SILVA 104 mothur NAST-aligned pyrotags with default values and the 'pos_var_bac' 0 filter, with local support based on the Shimodaira-Hasegawa test.

Krona javascript files for Lee et al. Microbial Response to SCP Production. html files draw from local copies of img and src Krona 2.3 files. All sequence counts have been aggregated across time points for each sample environment, listed below. Total sequences includes 1 additional control sample.

Legend:

In = Plant Influent Sample Port
Eq = Acidogenic Basin Mixed Liquor
Meth = Methanogenesis UASB Outfall
Aeb = Aerobic Basin Mixed Liquor
Out = Clarifier Outfall
Ob = Pilot Bioreactor Sample Port
SCP = Dried SCP Product

Aeb.1	4/30/09
Aeb.2A	4/30/09
Aeb.2B	4/30/09
Eq.5	1/30/08
Eq.1	3/19/08
Eq.3	3/19/09
Eq.42	4/30/09
In.4	1/30/08
In.1	3/19/08
In.3	4/30/09
Meth.4	1/30/08
Meth.1	3/19/08
Meth.22	4/30/09
Ob.1	3/19/08
Ob.7	4/8/08
Ob.2	9/10/08
Ob.3	1/6/09
Ob.4	2/16/09
Ob.52	3/19/09
Ob.62	4/30/09
Out.3	1/30/08
Out.1	3/19/08
Out.2	4/30/09
Scp.2	5/7/08
Scp.3	9/10/08
Scp.1	1/6/09
Scp.4	2/16/09
Scp.5	3/19/09
Scp.62	4/30/09

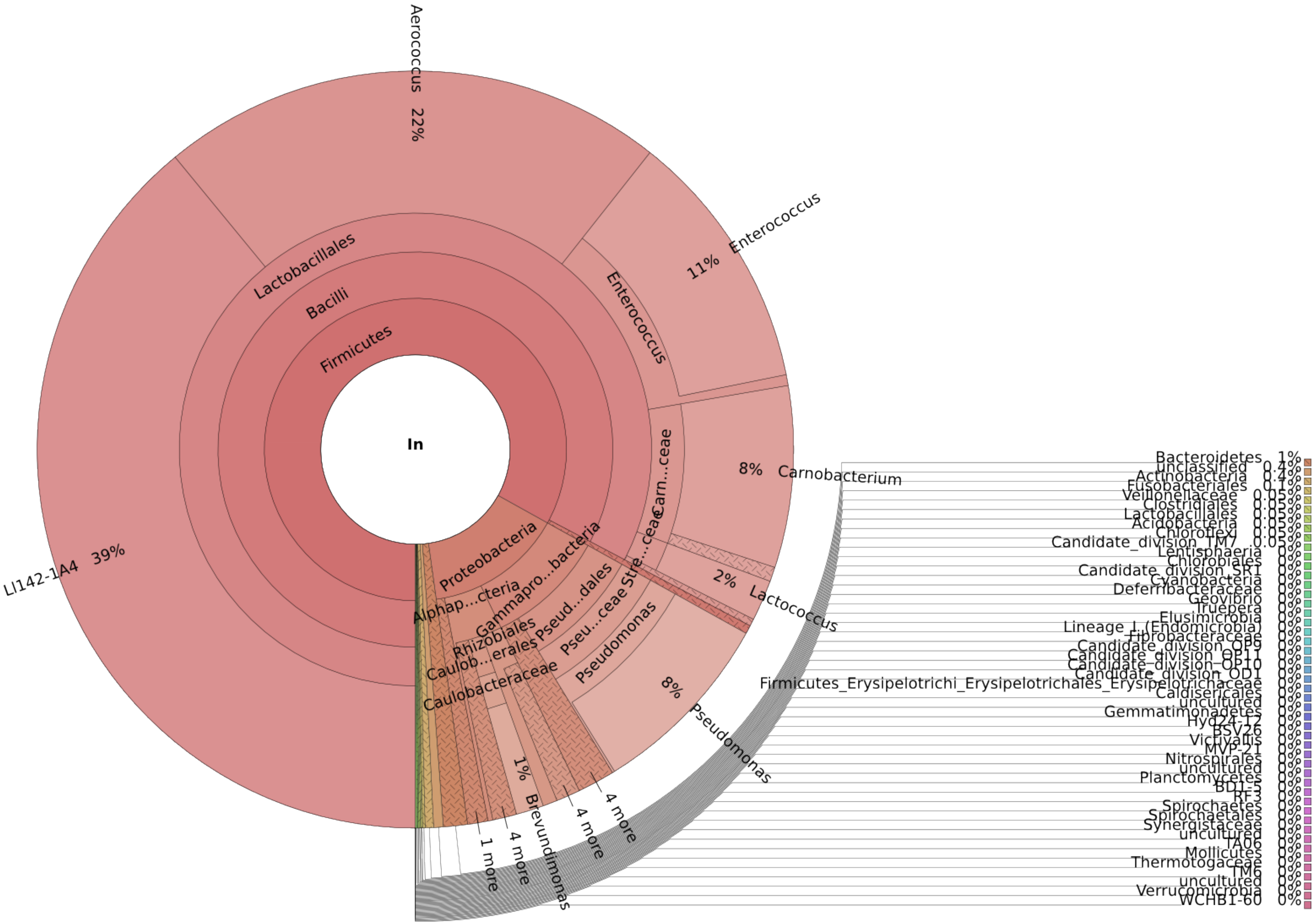

In
Firmicutes
Bacilli
Lactobacillales
Aerococcus 22%
Enterococcus
11% Enterococcus
Carn...ceae
8% Carnobacterium
Ste...ceae
2% Lactococcus
Ll142-1A4 39%
Proteobacteria
Gammapro...bacteria
Pseud...dales
Pseu...ceae
Pseudomonas
8% Pseudomonas
Alphap...cteria
Rhizobiales
Caulob...erales
Caulobacteraceae
1% Brevundimonas
1 more
4 more
4 more
4 more
Bacteroidetes 1%
unclassified 0.4%
Actinobacteria 0.4%
Fusobacteriales 0.1%
Veillonellaceae 0.05%
Clostridiales 0.05%
Lactobacillales 0.05%
Acidobacteria 0.05%
Chloroflexi 0.05%
Candidate_division_TM7 0.05%
Lentisphaeria 0%
Chlorobiales 0%
Candidate_division_SR1 0%
Cyanobacteria 0%
Deferribacteraceae 0%
Geovibrio 0%
Truepera 0%
Elusimicrobia 0%
Lineage_I_(Endomicrobia) 0%
Fibrobacteraceae 0%
Candidate_division_OP9 0%
Candidate_division_OP11 0%
Candidate_division_OP10 0%
Candidate_division_OD1 0%
Firmicutes_Erysipelotrichi_Erysipelotrichales_Erysipelotrichaceae 0%
Caldisericales 0%
uncultured 0%
Gemmatimonadetes 0%
Hyd24-12 0%
BSV26 0%
Victivallis 0%
MVP-21 0%
Nitrospirales 0%
uncultured 0%
Planctomycetes 0%
BD1-5 0%
RF3 0%
Spirochaetes 0%
Spirochaetales 0%
Synergistaceae 0%
uncultured 0%
TA06 0%
Mollicutes 0%
Thermotogaceae 0%
TM6 0%
uncultured 0%
Verrucomicrobia 0%
WCHB1-60 0%

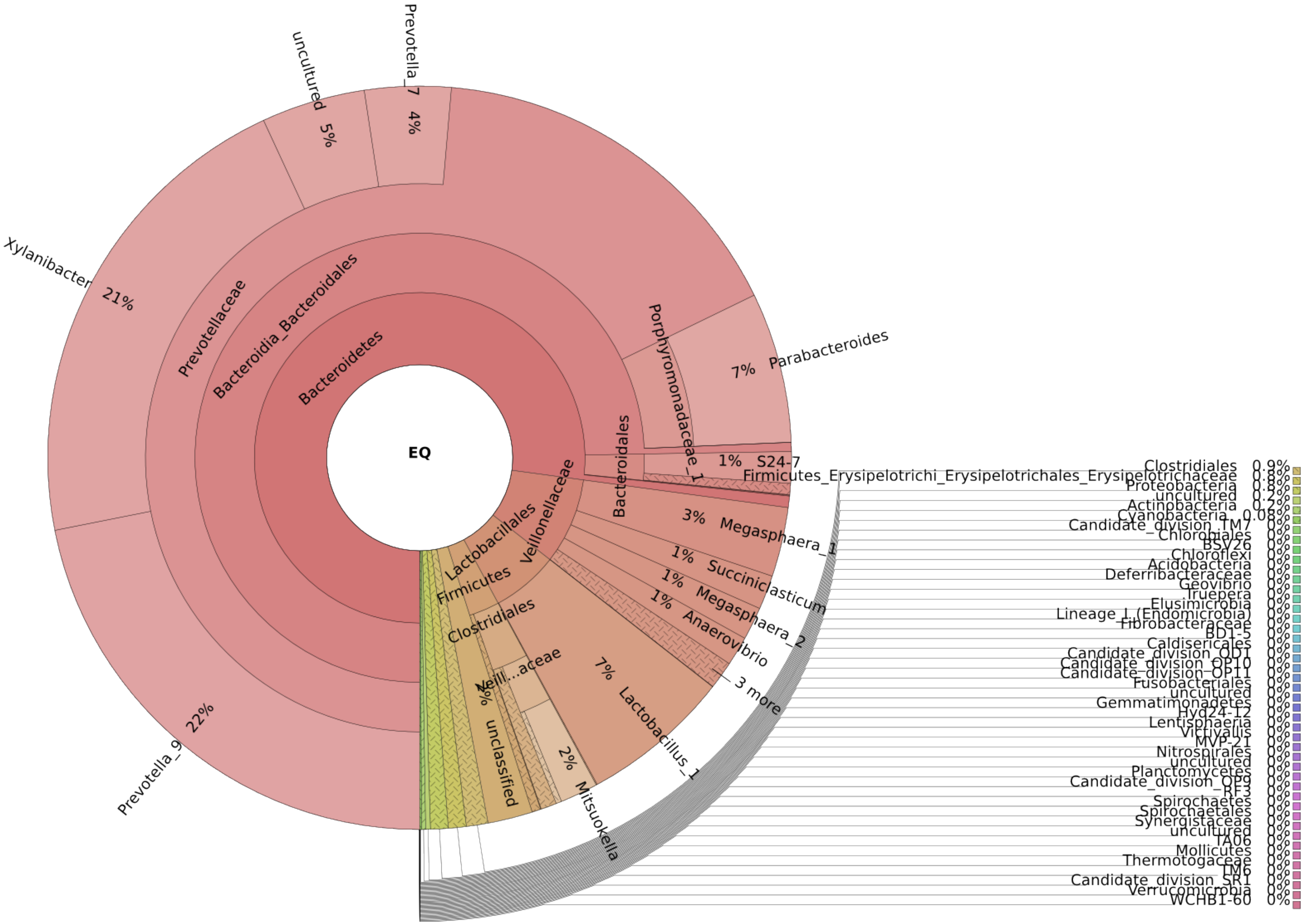
EQ
Bacteroidetes
Bacteroidia_Bacteroidales
Prevotellaceae
Xylanibacter 21%
uncultured 5%
Prevotella_7 4%
Prevotella_9 22%
Porphyromonadaceae_1
7% Parabacteroides
Bacteroidales
1% S24-7
Firmicutes_Erysipelotrichi_Erysipelotrichales_Erysipelotrichaceae
Veillonellaceae
3% Megasphaera_1
1% Succiniclasticum
1% Megasphaera_2
1% Anaerovibrio
3 more
Lactobacillales
Firmicutes
Clostridiales
Veill...aceae
unclassified
7% Lactobacillus_1
2% Mitsuokella
Clostridiales 0.9%
Proteobacteria 0.8%
uncultured 0.2%
Actinobacteria 0.2%
Cyanobacteria 0.08%
Candidate_division_TM7 0%
Chlorobiales 0%
BSV26 0%
Chloroflexi 0%
Acidobacteria 0%
Deferribacteraceae 0%
Geovibrio 0%
Truepera 0%
Elusimicrobia 0%
Lineage_I_(Endomicrobia) 0%
Fibrobacteraceae 0%
BD1-5 0%
Caldisericales 0%
Candidate_division_OD1 0%
Candidate_division_OP10 0%
Candidate_division_OP11 0%
Fusobacteriales 0%
uncultured 0%
Gemmatimonadetes 0%
Hyd24-12 0%
Lentisphaeria 0%
Victivallis 0%
MVP-21 0%
Nitrospirales 0%
uncultured 0%
Planctomycetes 0%
Candidate_division_OP9 0%
RF3 0%
Spirochaetes 0%
Spirochaetales 0%
Synergistaceae 0%
uncultured 0%
TA06 0%
Mollicutes 0%
Thermotogaceae 0%
TM6 0%
Candidate_division_SR1 0%
Verrucomicrobia 0%
WCHB1-60 0%

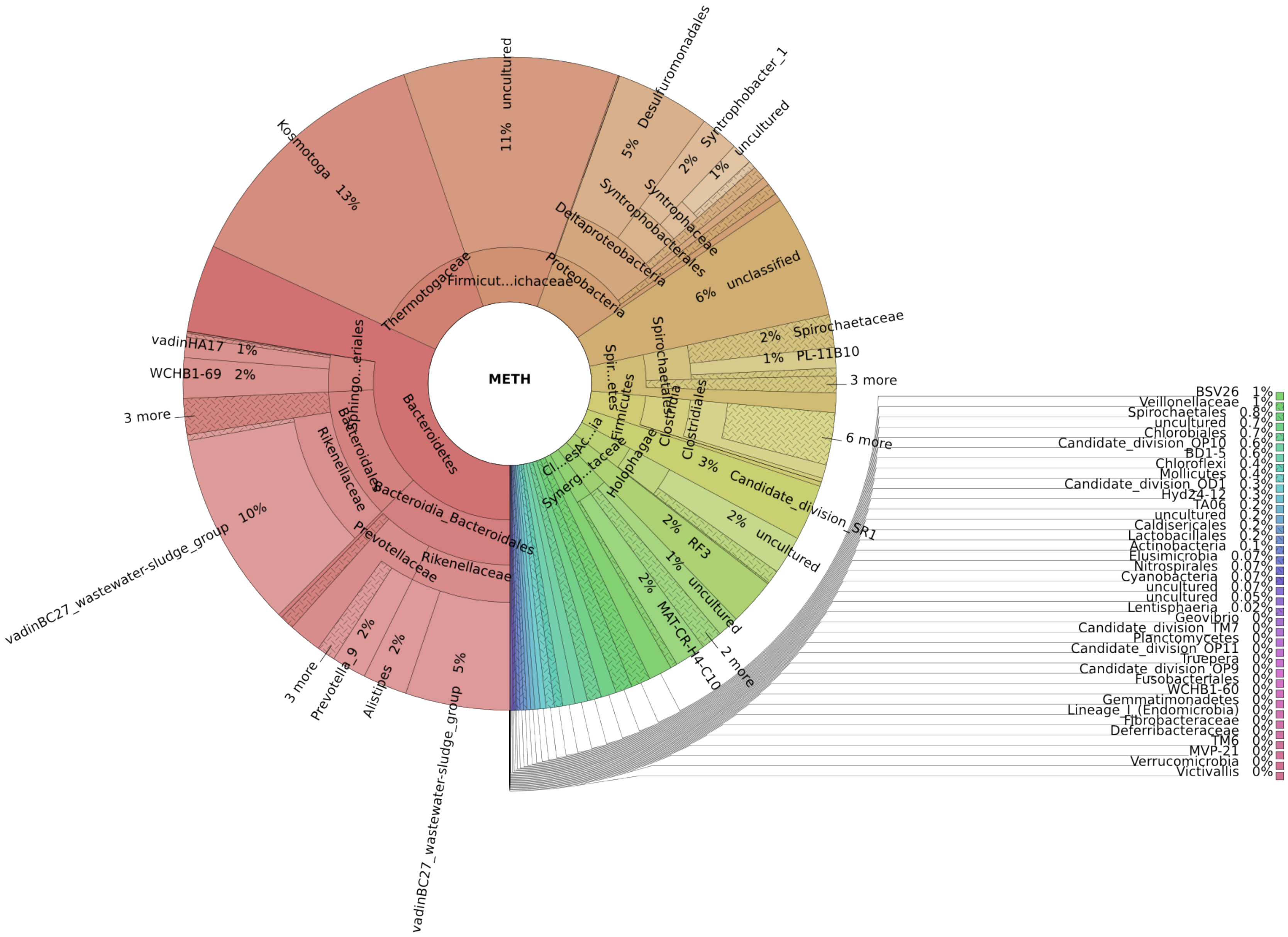

METH
uncultured 11%
Firmicut...ichaceae
Kosmotoga 13%
Thermotogaceae
Desulfuromonadales 5%
Syntrophobacter_1 2%
uncultured 1%
Syntrophaceae
Syntrophobacterales
Deltaproteobacteria
Proteobacteria
6% unclassified
2% Spirochaetaceae
1% PL-11B10
3 more
Spirochaetales
Spir...etes
Firmicutes
Clostridia
Clostridiales
6 more
3% Candidate_division_SR1
2% uncultured
2% RF3
1% uncultured
2% MAT-CR-H4-C10
2 more
Holophagae
Synerg...taceae
Cl...es
Ac...ia
Bacteroidetes
Bacteroidia_Bacteroidales
Bacteroidales
Sphingo...eriales
vadinHA17 1%
WCHB1-69 2%
3 more
Rikenellaceae
vadinBC27_wastewater-sludge_group 10%
Prevotellaceae
Rikenellaceae
3 more
Prevotella_9 2%
Alistipes 2%
vadinBC27_wastewater-sludge_group 5%
BSV26 1%
Veillonellaceae 1%
Spirochaetales 0.8%
uncultured 0.7%
Chlorobiales 0.7%
Candidate_division_OP10 0.6%
BD1-5 0.6%
Chloroflexi 0.4%
Mollicutes 0.4%
Candidate_division_OD1 0.3%
Hyd24-12 0.3%
TA06 0.2%
uncultured 0.2%
Caldisericales 0.2%
Lactobacillales 0.2%
Actinobacteria 0.1%
Elusimicrobia 0.07%
Nitrospirales 0.07%
Cyanobacteria 0.07%
uncultured 0.07%
uncultured 0.05%
Lentisphaeria 0.02%
Geovibrio 0%
Candidate_division_TM7 0%
Planctomycetes 0%
Candidate_division_OP11 0%
Truepera 0%
Candidate_division_OP9 0%
Fusobacteriales 0%
WCHB1-60 0%
Gemmatimonadetes 0%
Lineage_I_(Endomicrobia) 0%
Fibrobacteraceae 0%
Deferribacteraceae 0%
TM6 0%
MVP-21 0%
Verrucomicrobia 0%
Victivallis 0%

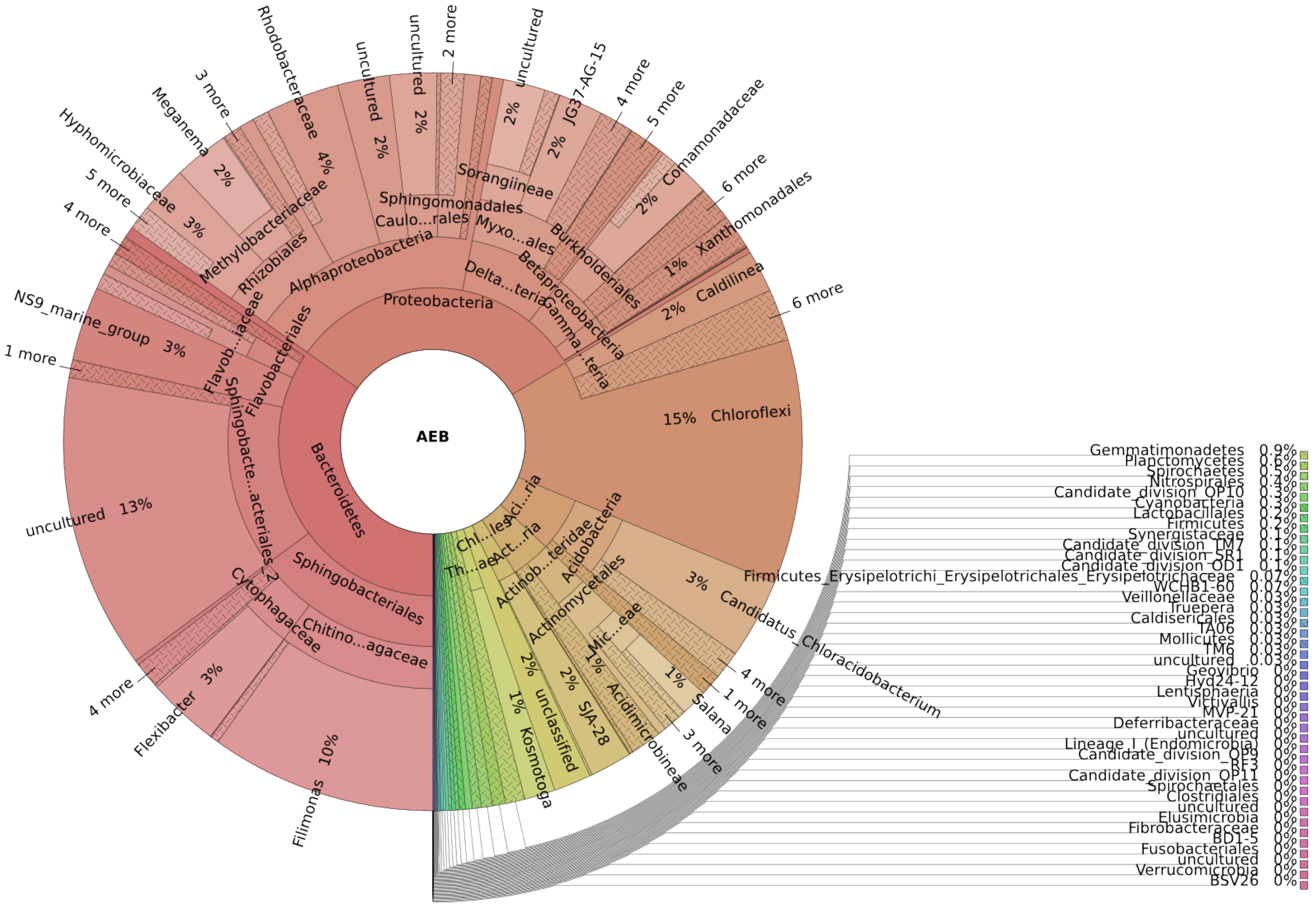

AEB
Proteobacteria
Alphaproteobacteria
Rhodobacteraceae 4%
uncultured 2%
uncultured 2%
2 more
Sphingomonadales
Caulo...rales
Rhizobiales
Methylobacteriaceae
Hyphomicrobiaceae 3%
Meganema 2%
3 more
5 more
4 more
Delta...teria
Myxo...ales
Sorangiineae
uncultured 2%
JG37-AG-15 2%
Betaproteobacteria
Burkholderiales
4 more
5 more
Comamonadaceae 2%
6 more
Gamma...teria
Xanthomonadales 1%
Caldilinea 2%
6 more
Chloroflexi 15%
Bacteroidetes
Flavobacteriales
Flavob...iaceae
NS9_marine_group 3%
1 more
Sphingobacte...acteriales_2
uncultured 13%
Sphingobacteriales
Cytophagaceae
Chitino...agaceae
4 more
Flexibacter 3%
Filimonas 10%
Acidobacteria
Candidatus_Chloracidobacterium 3%
4 more
1 more
Aci...ria
Act...ria
Actinob...teridae
Actinomycetales
Mic...eae 1%
Salana 1%
Acidimicrobineae
3 more
SJA-28 2%
Chl...les
Th...ae
unclassified 2%
Kosmotoga 1%
Gemmatimonadetes 0.9%
Planctomycetes 0.6%
Spirochaetes 0.5%
Nitrospirales 0.4%
Candidate_division_OP10 0.3%
Cyanobacteria 0.3%
Lactobacillales 0.2%
Firmicutes 0.2%
Synergistaceae 0.1%
Candidate_division_TM7 0.1%
Candidate_division_SR1 0.1%
Candidate_division_OD1 0.1%
Firmicutes_Erysipelotrichi_Erysipelotrichales_Erysipelotrichaceae 0.07%
WCHB1-60 0.07%
Veillonellaceae 0.03%
Truepera 0.03%
Caldisericales 0.03%
TA06 0.03%
Mollicutes 0.03%
TM6 0.03%
uncultured 0.03%
Geovibrio 0%
Hyd24-12 0%
Lentisphaeria 0%
Victivallis 0%
MVP-21 0%
Deferribacteraceae 0%
uncultured 0%
Lineage_I_(Endomicrobia) 0%
Candidate_division_OP9 0%
RF3 0%
Candidate_division_OP11 0%
Spirochaetales 0%
Clostridiales 0%
uncultured 0%
Elusimicrobia 0%
Fibrobacteraceae 0%
BD1-5 0%
Fusobacteriales 0%
uncultured 0%
Verrucomicrobia 0%
BSV26 0%

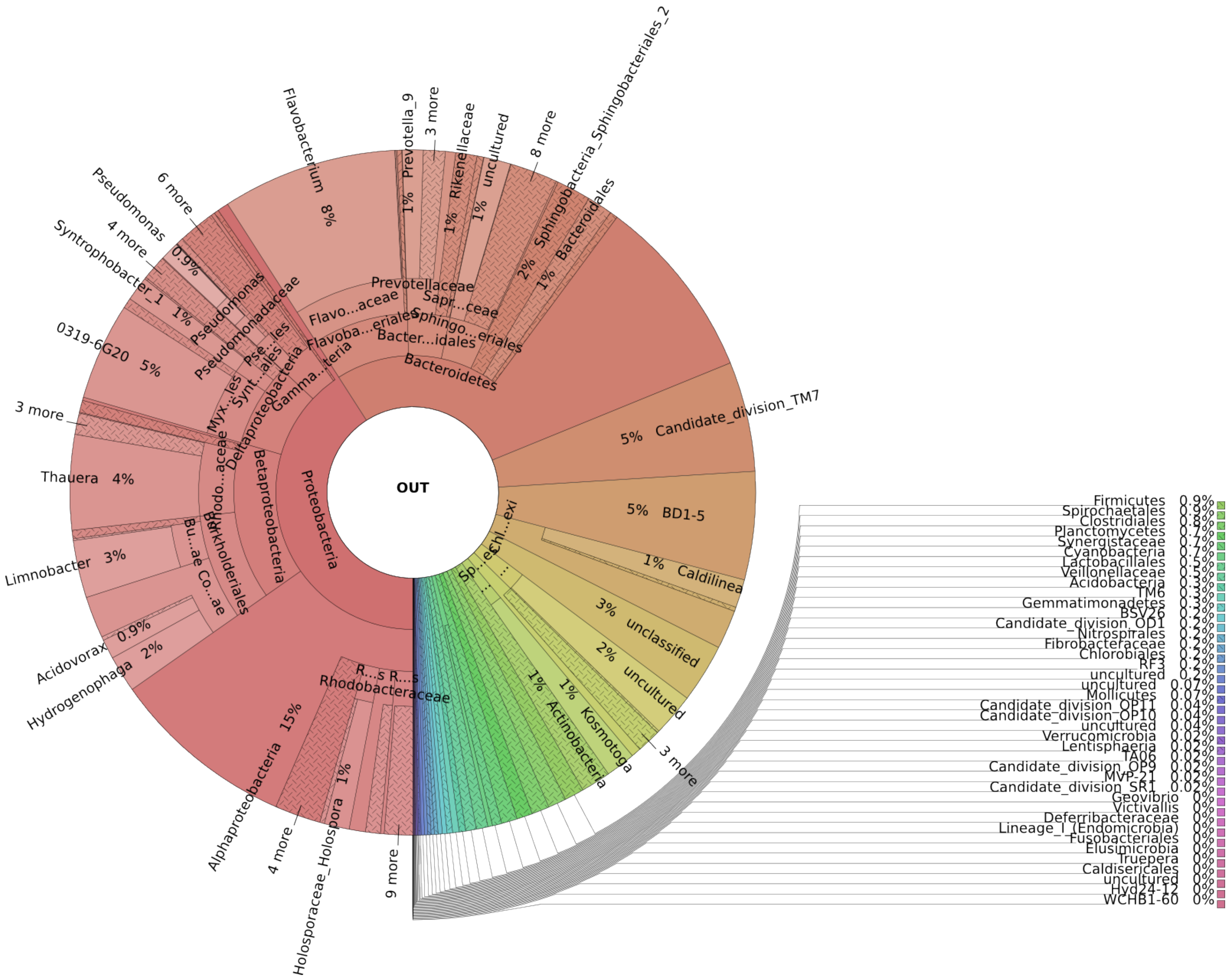
OUT
Proteobacteria
Betaproteobacteria
Burkholderiales
Rhodo...aceae
Thauera 4%
Limnobacter 3%
Bu...ae Co...ae
Acidovorax 0.9%
Hydrogenophaga 2%
3 more
Alphaproteobacteria 15%
Rhodobacteraceae
R...s R...s
4 more
Holosporaceae_Holospora 1%
9 more
Deltaproteobacteria
Myx...les
Synt...ales
0319-6G20 5%
Syntrophobacter_1 1%
Gamma...teria
Pse...les
Pseudomonadaceae
Pseudomonas 0.9%
4 more
6 more
Bacteroidetes
Bacter...idales
Flavoba...eriales
Flavo...aceae
Flavobacterium 8%
Prevotellaceae
Prevotella_9 1%
3 more
Sapr...ceae
Rikenellaceae 1%
uncultured 1%
Sphingo...eriales
8 more
Sphingobacteria_Sphingobacteriales_2 2%
Bacteroidales 1%
Candidate_division_TM7 5%
BD1-5 5%
Chl...exi
Caldilinea 1%
unclassified 3%
uncultured 2%
Sp...es
3 more
Kosmotoga 1%
Actinobacteria 1%
Firmicutes 0.9%
Spirochaetales 0.9%
Clostridiales 0.8%
Planctomycetes 0.7%
Synergistaceae 0.7%
Cyanobacteria 0.7%
Lactobacillales 0.5%
Veillonellaceae 0.5%
Acidobacteria 0.3%
TM6 0.3%
Gemmatimonadetes 0.3%
BSV26 0.2%
Candidate_division_OD1 0.2%
Nitrospirales 0.2%
Fibrobacteraceae 0.2%
Chlorobiales 0.2%
RF3 0.2%
uncultured 0.2%
uncultured 0.07%
Mollicutes 0.07%
Candidate_division_OP11 0.04%
Candidate_division_OP10 0.04%
uncultured 0.04%
Verrucomicrobia 0.02%
Lentisphaeria 0.02%
TA06 0.02%
Candidate_division_OP9 0.02%
MVP-21 0.02%
Candidate_division_SR1 0.02%
Geovibrio 0%
Victivallis 0%
Deferribacteraceae 0%
Lineage_I_(Endomicrobia) 0%
Fusobacteriales 0%
Elusimicrobia 0%
Truepera 0%
Caldisericales 0%
uncultured 0%
Hyd24-12 0%
WCHB1-60 0%

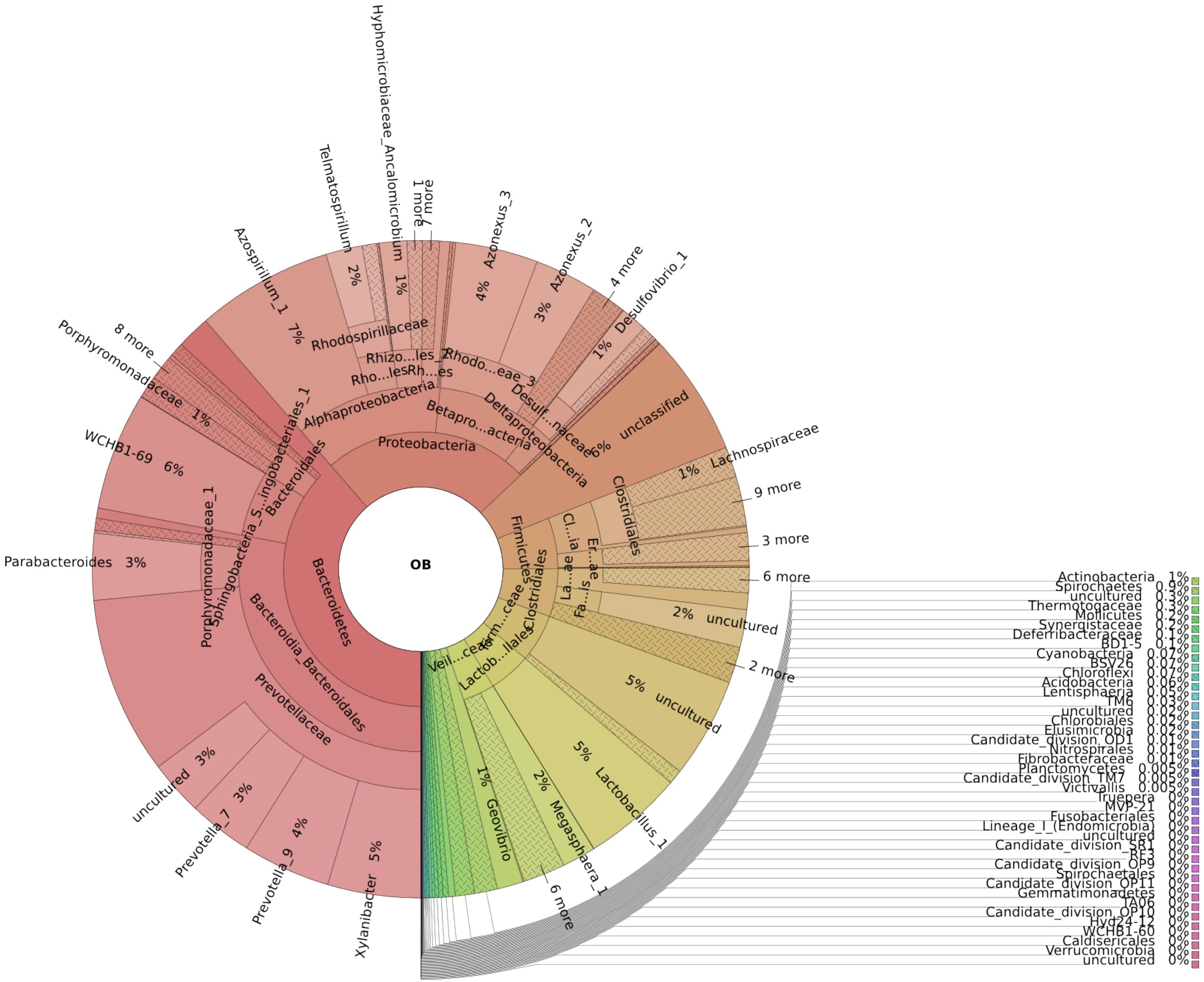

OB
Proteobacteria
Alphaproteobacteria
Rhodospirillaceae
Azospirillum_1 7%
Telmatospirillum 2%
Hyphomicrobiaceae_Ancalomicrobium 1%
1 more
7 more
Rhizo...les_2
Rho...les
Rh...es
Betapro...acteria
Rhodo...eae_3
Azonexus_3 4%
Azonexus_2 3%
4 more
Deltaproteobacteria
Desulf...naceae
Desulfovibrio_1 1%
unclassified 6%
Firmicutes
Clostridiales
Cl...ia
Er...ae
La...ae
Fa...is
Lachnospiraceae 1%
9 more
3 more
6 more
uncultured 2%
2 more
uncultured 5%
Lactobacillus_1 5%
Lactob...llales
Firm...ceae
Veil...ceae
Megasphaera_1 2%
6 more
Geovibrio 1%
Bacteroidetes
Bacteroidia_Bacteroidales
Prevotellaceae
Xylanibacter 5%
Prevotella_9 4%
Prevotella_7 3%
uncultured 3%
Sphingobacteria_S...ingobacteriales_1
Bacteroidales
Porphyromonadaceae_1
Parabacteroides 3%
WCHB1-69 6%
Porphyromonadaceae 1%
8 more
Actinobacteria 1%
Spirochaetes 0.9%
uncultured 0.3%
Thermotogaceae 0.3%
Mollicutes 0.2%
Synergistaceae 0.2%
Deferribacteraceae 0.1%
BD1-5 0.1%
Cyanobacteria 0.07%
BSV26 0.07%
Chloroflexi 0.07%
Acidobacteria 0.06%
Lentisphaeria 0.05%
TM6 0.03%
uncultured 0.02%
Chlorobiales 0.02%
Elusimicrobia 0.02%
Candidate_division_OD1 0.01%
Nitrospirales 0.01%
Fibrobacteraceae 0.01%
Planctomycetes 0.005%
Candidate_division_TM7 0.005%
Victivallis 0.005%
Truepera 0%
MVP-21 0%
Fusobacteriales 0%
Lineage_I_(Endomicrobia) 0%
uncultured 0%
Candidate_division_SR1 0%
RF3 0%
Candidate_division_OP9 0%
Spirochaetales 0%
Candidate_division_OP11 0%
Gemmatimonadetes 0%
TA06 0%
Candidate_division_OP10 0%
Hyd24-12 0%
WCHB1-60 0%
Caldisericales 0%
Verrucomicrobia 0%
uncultured 0%

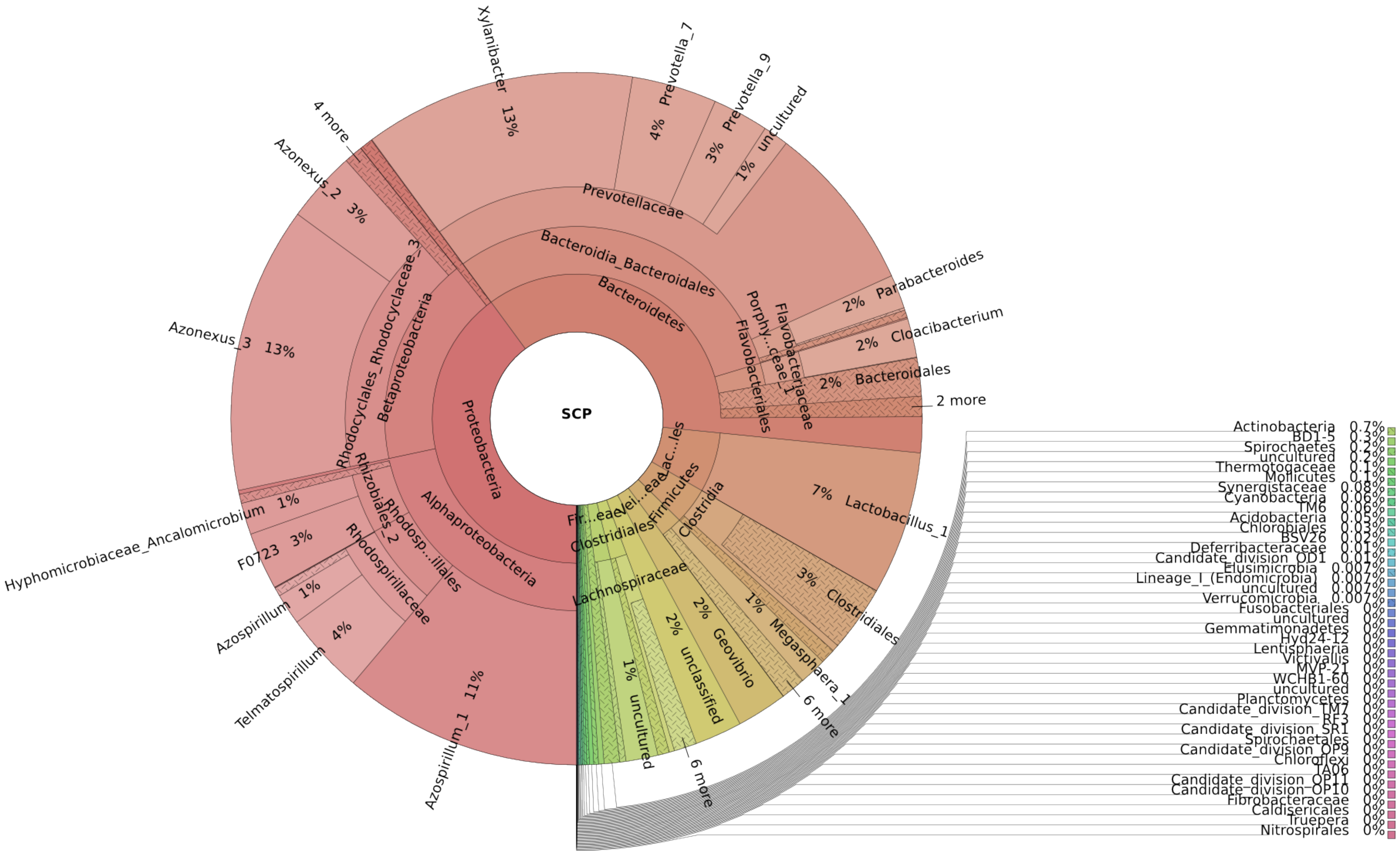
SCP
Bacteroidetes
Bacteroidia_Bacteroidales
Prevotellaceae
Xylanibacter 13%
Prevotella_7 4%
Prevotella_9 3%
uncultured 1%
Parabacteroides 2%
Cloacibacterium 2%
Bacteroidales 2%
2 more
Flavobacteriales
Flavobacteriaceae
Porphy...ceae_1
Proteobacteria
Betaproteobacteria
Rhodocyclales_Rhodocyclaceae_3
Azonexus_3 13%
Azonexus_2 3%
4 more
Alphaproteobacteria
Rhizobiales_2
Rhodosp...illales
Rhodospirillaceae
Hyphomicrobiaceae_Ancalomicrobium 1%
F0723 3%
Azospirillum 1%
Telmatospirillum 4%
Azospirillum_1 11%
Firmicutes
Lac...les
Lactobacillus_1 7%
Clostridia
Clostridiales 3%
Megasphaera_1 1%
6 more
Geovibrio 2%
unclassified 2%
uncultured 1%
6 more
Lachnospiraceae
Clostridiales
Fir...eae
vei...eae
Actinobacteria 0.7%
BD1-5 0.3%
Spirochaetes 0.2%
uncultured 0.2%
Thermotogaceae 0.1%
Mollicutes 0.1%
Synergistaceae 0.08%
Cyanobacteria 0.06%
TM6 0.06%
Acidobacteria 0.03%
Chlorobiales 0.03%
BSV26 0.02%
Deferribacteraceae 0.01%
Candidate_division_OD1 0.01%
Elusimicrobia 0.007%
Lineage_I_(Endomicrobia) 0.007%
uncultured 0.007%
Verrucomicrobia 0.007%
Fusobacteriales 0%
uncultured 0%
Gemmatimonadetes 0%
Hyd24-12 0%
Lentisphaeria 0%
Victivallis 0%
MVP-21 0%
WCHB1-60 0%
uncultured 0%
Planctomycetes 0%
Candidate_division_TM7 0%
RF3 0%
Candidate_division_SR1 0%
Spirochaetales 0%
Candidate_division_OP9 0%
Chloroflexi 0%
TA06 0%
Candidate_division_OP11 0%
Candidate_division_OP10 0%
Fibrobacteraceae 0%
Caldisericales 0%
Truepera 0%
Nitrospirales 0%
